# Effective Quantum Theory of EXAFS in a Dissipative Liquid-Phase Medium

**DOI:** 10.1021/acs.jpcb.5c06230

**Published:** 2025-12-18

**Authors:** Mei Bai, Robin Santra, Sang-Kil Son, Henning Kirchberg, Michael Thorwart

**Affiliations:** † I. Institut für Theoretische Physik, 14915 Universität Hamburg, Notkestraße 9, 22607 Hamburg, Germany; ‡ The Hamburg Centre for Ultrafast Imaging, Universität Hamburg, Luruper Chaussee 149, 22761 Hamburg, Germany; § Center for Free-Electron Laser Science CFEL, Deutsches Elektronen-Synchrotron DESY, Notkestr. 85, 22607 Hamburg, Germany; ∥ Department of Microtechnology and Nanoscience, 11248 Chalmers University of Technology, Kemivägen 9, 41296 Göteborg, Sweden

## Abstract

The extended X-ray absorption fine structure (EXAFS) spectroscopy is a powerful tool to determine the microscopic structure in the vicinity of a probe atom or molecule embedded in a host material. For absorbing atoms dissolved in a liquid, the disordered nature of the host poses challenges for the theoretical calculation of the EXAFS spectrum, especially when strong inelastic energy and momentum transfer between the photoelectron and the solvent occurs. We formulate here an effective quantum theory of EXAFS that is based on the use of an accurately parametrized complex dielectric function of the solvent, illustrated here for the case of water. We derive an effective complex self-energy within the GW approximation to determine the EXAFS signal within a single-scattering approach. To verify the approach, we recover the results for the inelastic mean free path of a photoelectron in water, as known in the literature. In addition, we apply this effective approach to the case of single bromide and chloride atoms dissolved in water and show that the theoretical results match available experimental data. Through advanced FEFF simulations, which include accurate multiple-scattering effects, we conclude that the contribution of the single-scattering processes is dominant. We show that a key role is played by the dielectric environment.

## Graphical abstract

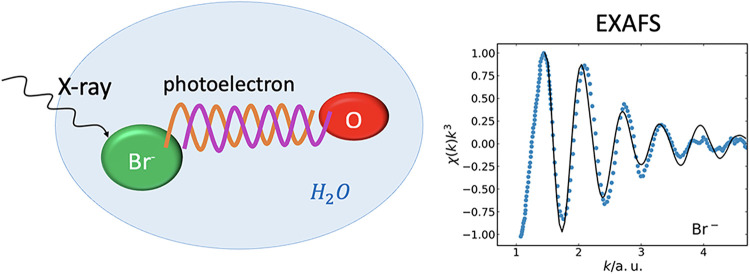

## 1 Introduction

X-ray absorption spectroscopy has become a well-established technique for determining the local microscopic environment of a selected analyte atom in the gas, liquid, or solid phase.^1–7^ Extended X-ray absorption fine-structure (EXAFS) spectroscopy^8,9^ provides fine details of the partial pair distribution functions of atoms surrounding the absorbing analyte atom, such as distances, mean square deviations in distances, or coordination numbers. For applications of EXAFS to liquids, see, e.g., refs 10–13 . In EXAFS spectroscopy, the photon energy of an incident monochromatic X-ray beam is scanned through an absorption edge of a selected atomic species. A photoelectron with a well-defined kinetic energy and, thus, a well-defined de Broglie wavelength is emitted and coherently scattered by the neighboring atoms, typically within the first few coordination shells around the analyte. The coherently scattered photoelectron interferes with the incoming wave. Depending on the kinetic energy, the interference is constructive or destructive, which leads to an energy-dependent modulation of the effective absorption cross section of the absorber atom, which is visible in the oscillatory EXAFS spectrum.

The theoretical description of EXAFS is by now highly quantitative^14^ and naturally requires a careful treatment of the quantum many-body dynamics. An excellent and highly quantitative tool developed over the last decades is FEFF.^14–17^ It is a numerical code based on real-space Green’s functions for simulating various X-ray spectroscopies, taking into account multiple-scattering events and quantum many-body effects. The general strategy is a reduction to independent-electron pictures with correlations treated effectively in terms of various inelastic losses and self-energy shifts due to electronic interactions, molecular vibrations and structural disorder. Because the latter is inherently strong in liquids, advanced numerical atomistic simulations with extended sampling over many molecular configurations are required,^18–22^ which still remain challenging. For regular lattices in the solid state, the calculation of EXAFS spectra can exploit the periodicity of the lattice arrangements of the scattering atoms. For structureless solvents in the liquid state, this advantage cannot be used. Then, typically, atomistic simulations have been applied to determine the geometric arrangement of many atoms consisting of the absorbing atom and many scattering atoms.^22^ This poses numerical challenges for the calculation of EXAFS spectra in liquids still today.

FEFF is able to include inelastic processes due to a dielectric environment quantitatively^23^ by numerically sampling a continuous form of a phenomenological dielectric loss function by a finite number of supporting points each of which is treated in the sense of a plasmon pole in the GW approximation of the self-energy of the photoelectron. Their contributions are summed up until numerical convergence is reached.

In EXAFS experiments, the possibility of photoelectron interference is temporally limited by the lifetime of the core hole left behind after inner-shell photoionization. This is of the order of a few hundred attoseconds to at most 1 fs.^24^ When the photoelectron is formed in a liquid solvent host, the response of the solvent on this ultrafast electronic time scale becomes relevant. Clearly, when the solvent is characterized by its dielectric function, its response properties in the optical sector in the energy regime of eV and of wave numbers in the regime of a few Å^–1^ are of critical importance. In contrast, the response of the solvent on much longer nuclear time scales or in the regime of long wavelengths is less relevant and mostly negligible for EXAFS.

In this work, we formulate an effective quantum dissipative theory of EXAFS spectroscopy on systems in the liquid phase by describing the self-interfering photoelectron at the level of single scattering from individual solvent atoms immersed in a continuous dielectric solvent. The photoelectron interacts with the high-energy and small-wavenumber modes of a continuous dielectric solvent, which modifies its interference properties by dephasing and by inducing an energy shift given by the solvent reorganization energy. We describe the polarizable solvent in terms of its frequency- and wavenumber-dependent complex permittivity. To be specific, we use an effective parametrization^25–27^ of the complex permittivity of water measured in experiments.^28–31^ The complex permittivity enters in an effective self-energy, also known as optical potential, of the photoelectron, thereby affecting the photoelectron phase and energy. The permittivity describes in an effective way energy and momentum transfer between the photoelectron and the dielectric continuum. This approach is not restricted to the case of water as a solvent as studied in the present work, but is applicable whenever a complex permittivity of the solvent is available in the regime of energies in the eV range and of wave numbers in the Å^–1^ range. Thereby, the dielectric permittivity can be used in the form of an effective, parametrized function, or, of an array of data stemming, e.g., from measurements or from numerical simulations. The dielectric function provides a macroscopic description of solvent polarization on length scales much larger than or, at most, comparable to intermolecular distances. It neglects details on the intramolecular length scales of the solvent molecules such as local field effects and specific solute–solvent short-range interactions. It assumes linear, homogeneous, and isotropic response, which can break down under strong fields. Consequently, this approach in terms of the dielectric function should be viewed as an effective approximation rather than a fully microscopic representation of solvent dynamics, especially in ultrafast nonequilibrium processes like the details of photoelectron emission.

The approach developed in this work relies on single-scattering events (in principle, there is no obstacle to generalizing this approach to include multiple-scattering events), albeit inelastic losses of the photoelectron scattering from the solvent are included accurately. Through comparison with FEFF,^14–17^ we assess the role of multiple scattering in liquid-phase EXAFS. As it turns out, the role of inelastic energy and momentum transfer in single scattering is more important than the contributions from multiple-scattering events. The single-scattering approximation simplifies EXAFS to pairwise linear interactions between the absorbing solute and scattering solvent molecule, but neglects angular effects and multiple-paths interference effects that could be crucial under peculiar circumstances for accurately describing ordered or very complex molecular structures.

Photoabsorption from the electronic ground state of an absorbing atom in EXAFS leads to the formation of a particle-hole pair. In general, this excited state couples to higher excitation classes, such as two-particle-two-hole excitations, via Coulomb interaction. Yet, a reduced description in terms of an effective Hamiltonian living in the one-particle-one-hole subspace is desirable. Indeed, an effective approach can be justified by the fact that the coupling to the transverse part of the electromagnetic field is mediated by a one-body operator. In standard EXAFS spectroscopy, the one-photon absorption signal is dominated by the properties of one effective particle–hole pair. However, there is a price to pay: the resulting effective Hamiltonian reduced to the one-particle-one-hole subspace becomes non-Hermitian, with a spectrum of complex, as opposed to real, eigenvalues.

In particular for the inner-shell photoionization in the condensed phase, there are two physical reasons which lead to a non-Hermitian effective Hamiltonian. First, the inner-shell hole is metastable, with typical lifetimes of the order of 1 fs or shorter. Second, the generated photoelectron may undergo inelastic collisions with atoms in the surroundings and may, in particular, lead to electron impact ionization. Hence, both the particle and the hole have a finite lifetime, causing a nonzero imaginary part of the associated energies. The approach which we develop in the present work takes both of these finite-lifetime effects into consideration.

The structure of the paper is as follows: In section 2, we briefly summarize the theoretical methods employed in this work. (Details are provided in the Supporting Information (SI).) Our results are shown and discussed in section 3. Specifically, we analyze the impact of the dielectric solvent on the EXAFS signal in section 3.1; in section 3.2, we provide a comparison to experimental data and FEFF calculations. Conclusions are presented in section 4.

## 2 Theoretical Methods

As explained in SI section S1, the X-ray absorption cross section may be written as

1 σabs=4παωx∑σ∫d3k∫d3k′Im{⟨Ψ0|ĉiσ†ĉk⃗σ1Ĥmatter−E0−ωx−iζĉk⃗′σ†ĉiσ|Ψ0⟩(k⃗·ϵ⃗*)⟨k⃗|i⟩*(k⃗′·ϵ⃗)⟨k⃗′|i⟩}

This expression is given in atomic units (a.u.), as are all other equations in this section. In eq 1, α is the fine-structure constant; ω_*x*_ is the X-ray photon energy; σ is the spin projection of the photoelectron; *k⃗* and *k⃗*′ refer to possible momentum states of the photoelectron; *Ĥ*_matter_ is the many-electron Hamiltonian; Ψ_0_ is the initial many-electron state of the system being investigated; *E*_0_ is the eigenenergy of *Ĥ*_matter_ in the state Ψ_0_; ζ > 0 is a positive infinitesimal; the annihilation operator *ĉ*_*iσ*_ creates a hole (annihilates an electron) in the *i*th initially occupied inner-shell orbital with spin projection σ, whereas the creation operator *ĉ*_*k⃗*′σ_^†^ creates an electron in the plane-wave state (*k⃗*′, σ); ϵ⃗ is the X-ray polarization vector; and

2 $$\left\langle \mathrm{k⃗}\right|i\rangle =\frac{1}{{\left(2\pi \right)}^{3/2}}\int d^{3}x\;e^{-i\mathrm{k⃗}\cdot \mathrm{x⃗}}\varphi_{i}\left(\mathrm{x⃗}\right)$$

is the overlap of the core orbital φ_*i*_(*x⃗*) with the plane-wave photoelectron state with momentum *k⃗*. Because of the spatial localization of φ_*i*_(*x⃗*), the overlap ⟨*k⃗*|*i*⟩ is at most weakly dependent on the chemical environment. If φ_*i*_(*x⃗*) is determined through a purely atomic mean-field calculation, ⟨*k⃗*|*i*⟩ is completely independent of the environment.

The electronic many-body physics associated with the absorption of an X-ray photon is encapsulated in the object

3 $$\left\langle \Psi_{0}\right|{ĉ}_{i\sigma }^{†}{ĉ}_{\mathrm{k⃗}\sigma }\frac{1}{{Ĥ}_{\mathrm{matter}}-E_{0}-\omega_{x}-i\zeta }{ĉ}_{{\mathrm{k⃗}}^{\prime }\sigma }^{†}{ĉ}_{i\sigma }\left|\Psi_{0}\right\rangle$$

which, up to a sign, may be interpreted as a one-particle Green’s function^32^ for the reference state *ĉ*_*iσ*_ |Ψ_0_⟩. Based on this observation (see SI section S2 for more details), we replace this object with the one-particle Green’s function

4 $$-G_{\mathrm{k⃗}\mathrm{k⃗}\prime }\left(E=\omega_{x}-I_{i}\right)=\left\langle \mathrm{k⃗}\right|\frac{1}{-\frac{1}{2}{\mathrm{\nabla ⃗}}^{2}+V_{\mathrm{sc}}+M\left(E=\omega_{x}-I_{i}\right)-i\frac{\Gamma_{i}}{2}-\left(\omega_{x}-I_{i}\right)}\left|{\mathrm{k⃗}}^{\prime }\right\rangle$$

Here, *I*_*i*_ is the binding energy (ionization potential) of the core orbital considered, so that

5 $$E=\omega_{x}-I_{i}$$

is the *nominal* kinetic energy of the photoelectron, i.e., the kinetic energy the photoelectron would have if it were immediately ejected into vacuum and did not find itself in a condensed-phase environment. The operator

6 $$-\frac{1}{2}{\mathrm{\nabla ⃗}}^{2}+V_{\mathrm{sc}}+M\left(E=\omega_{x}-I_{i}\right)$$

is an effective one-electron Hamiltonian for the photoelectron. On the one hand, the photoelectron interacts with the static mean-field potential

7 $$V_{\mathrm{sc}}\left(\mathrm{x⃗}\right)=\sum_{n}V_{n}\left(|\mathrm{x⃗}-{\mathrm{R⃗}}_{n}|\right)$$

which is assumed to include exchange effects in a local approximation and to be a sum over individual, spherically symmetric atomic contributions *V*_*n*_(|*x⃗* – *R⃗*_*n*_|), *R⃗*_*n*_ being the position of the *n*th nucleus in the system. On the other hand, the many-body physics not described by *V*_sc_ is captured by the photoelectron self-energy $M\left(E=\omega_{x}-I_{i}\right)$ . Finally, Γ_*i*_ in eq 4 is the decay width of the inner-shell hole.

Treating *V*_sc_ as a perturbation, expanding the photoelectron Green’s function in eq 4 through first order in that perturbation (see SI section S2), and inserting eq 7, the X-ray absorption cross section in eq 1 goes over into

8 σabs=−8παωx∫d3k∫d3k′Im{[G(0)(k,E)δ(k⃗−k⃗′)+G(0)(k,E)∑n⟨k⃗|Vn|k⃗′⟩G(0)(k′,E)](k⃗·ϵ⃗*)⟨k⃗|i⟩*(k⃗′·ϵ⃗)⟨k⃗′|i⟩}

Here

9 $$G^{\left(0\right)}\left(k,E\right)=\frac{1}{E-\left[\left(k^{2}/2\right)+M\left(k,E\right)-i\left(\Gamma_{i}/2\right)\right]}$$

is the zeroth-order Green’s function employed in this framework. Note that we include the photoelectron self-energy in $G^{\left(0\right)}\left(k,E\right)$ . The $M\left(E=\omega_{x}-I_{i}\right)$ in eq 4 is an operator, whereas the M(k,E) appearing in eq 9 is a matrix element of that operator in the momentum representation. We assume that, as far as the self-energy is concerned, the system is translationally invariant and isotropic, which is the reason why the self-energy in eq 9 is diagonal in the momentum representation and depends only on the magnitude *k* = |*k⃗*| of the photoelectron momentum (in addition to E ).

Equation 8 has an intuitive interpretation. In the expression of eq 7 for the scattering potential, the term with *n* = 0 refers to the parent atom of the photoelectron. We then see that for an *n* ≠ 0, the first-order term in eq 8 directly reflects the usual EXAFS picture: Photoabsorption in orbital *i* produces a photoelectron in the plane-wave state *k⃗*′, which is encoded in the term (*k⃗*′·ϵ⃗) ⟨*k⃗*′|*i*⟩. The “free” propagation in this state is described by $G^{\left(0\right)}\left(k^{\prime },E\right)$ . Then, scattering off the neighboring atom at *R⃗*_*n*_ occurs. This causes the photoelectron to undergo a transition from momentum *k⃗*′ to momentum *k⃗*, giving rise to the transition matrix element ⟨*k⃗*|*V*_*n*_|*k⃗*′⟩. Afterward, free propagation in this state is described by the propagator $G^{\left(0\right)}\left(k,E\right)$ . Interference with the pathway in which the photoelectron is produced directly with momentum *k⃗* is encoded in the factor (*k⃗*·ϵ⃗ *) ⟨*k⃗*|*i*⟩*.

As explained in more detail in SI sections S2 and S3, we use the GW-approximated self-energy:

10 $$M\left(k,E\right)=4\pi i\int \frac{d^{3}q}{{\left(2\pi \right)}^{3}}\;\frac{1}{{\mathrm{q⃗}}^{2}}\int_{0}^{\infty }\frac{d\omega }{2\pi }\;e^{-i\delta \omega }\frac{\Theta \left(\left|\mathrm{k⃗}-\mathrm{q⃗}\right|-k_{F}\right)}{E-\omega -\left({\left(\mathrm{k⃗}-\mathrm{q⃗}\right)}^{2}/2\right)+i\delta }\;\frac{1}{\varepsilon \left(\omega ,q\right)}$$

In this expression, *k*_F_ is the Fermi momentum of the homogeneous medium in which the photoelectron propagates, δ is a positive infinitesimal, and *ε*(ω, *q*) is the dielectric function of the medium. Physically, the self-energy in eq 10 describes how the photoelectron with momentum *k⃗* is inelastically scattered in the medium. In such a scattering event, the photoelectron transfers momentum *q⃗* and energy ω to the medium. Thus, after the collision, the photoelectron has momentum *k⃗* – *q⃗* and energy E−ω . The Heaviside function indicates that the photoelectron can only be scattered into states that are not already occupied.

The response of the medium to the impact of the photoelectron is captured by the dielectric function, which we parametrize using a Drude–Lorentz-type expression

11 $$\varepsilon \left(\omega ,q\right)=\frac{\omega_{0}^{2}\left(q\right)-\omega^{2}-i\omega \gamma \left(q\right)+\omega_{p}^{2}\left(q\right)}{\omega_{0}^{2}\left(q\right)-\omega^{2}-i\omega \gamma \left(q\right)}$$

We determine the *q*-dependent fit parameters ω_0_(*q*), γ­(*q*), and ω_*p*_(*q*) through comparison with the parametrization utilized in refs 27,28 . (see SI section S3). The advantage of the dielectric function we employ is that it captures the response properties of water quite accurately and that the ω integral in eq 10 can be evaluated analytically, thereby reducing the dimensionality of the integral that remains to be solved numerically. The integrations required are discussed in SI section S4. In SI section S5, we present a validation of the self-energy employed, based on a calculation of the inelastic mean free path of an electron in water. The relation to the common plasmon-pole approximation to the dielectric function is discussed in SI section S3.3.

The quantity of primary interest in EXAFS is the relative change, χ, of the X-ray absorption cross section caused by the presence of atomic scatterers in the environment of the absorbing atom. χ is a function of the nominal photoelectron kinetic energy $E=\omega_{x}-I_{i}$ (eq 5). Traditionally, χ is plotted as a function of the nominal photoelectron momentum

12 $$k=\sqrt{2E}=\sqrt{2\left(\omega_{x}-I_{i}\right)}$$

By evaluating the integrals appearing in eq 8 and making a few additional assumptions laid out in SI sections S6 and S7, we arrive at the following working equation for χ:

13 χ=∑n≠01Rn2Im{ei(2k̃Rn+π)fn(1)ei(ϕabs+ϕback)[k̃2/(Zeff2+k̃2)4]}Re{k̃3/(Zeff2+k̃2)4}

In this expression, the sum runs over all atoms in the environment of the parent atom of the photoelectron, *R*_*n*_ is the distance of the *n*th atom from the atom absorbing the X-ray photon, and *Z*_eff_ is the effective nuclear charge experienced by a K-shell electron in the absorbing atomic species. We focus in this work on K-shell photoionization, which is evident in eq 13 from the phase shift of π characteristic of a p-wave photoelectron.

Equation 13 rests on the *single-scattering* approximation, i.e., a photoelectron ejected from the X-ray absorber (the atom with index *n* = 0) may be backscattered to that parent atom from any atom in the environment of the X-ray absorber (any atom with *n* ≠ 0) and may then interfere with itself. Thus, in the single-scattering approximation, the index *n* ≠ 0 enumerates not only all atoms in the environment, but also all possible scattering pathways available. *Multiple-scattering* events, such as photoelectron scattering from one atom in the environment to another atom in the environment and then back to the parent atom, are not taken into consideration.

The central quantity appearing in eq 13 is the complex-valued momentum

14 $$\mathrm{k̃}\left(E\right)=\sqrt{2\left(E-M\left(\sqrt{2E},E\right)+i\frac{\Gamma_{i}}{2}\right)}$$

It is through *k̃* that the dissipative properties of the environment, encoded in the self-energy $M\left(\sqrt{2E},E\right)$ , and the decay of the core hole, through the decay width Γ_*i*_, enter in the EXAFS signal. *f*_*n*_^(1)^ in eq 13 is the amplitude for backscattering of the photoelectron from the *n*th atom, in the Born approximation, and is a function of *k̃*. We compute *f*_*n*_^(1)^ for each atomic species in the environment of the absorbing atom using XATOM,^33,34^ which is freely available to users through XRAYPAC.^35^ We also employ XATOM to compute nonperturbatively the phase shift ϕ_back_ associated with photoelectron backscattering from the *n*th atom, and the phase shift ϕ_abs_ connected to the influence of the atomic potential of the parent atom on the photoelectron.

## 3 Results and Discussion

In this section, we employ the model presented in section 2 to compute the Br and Cl K-shell EXAFS signals for Br^–^ and Cl^–^ dissolved in liquid water, focusing primarily on bromine. In addition to the atomic positions (more on this below); the dielectric function of water (SI section S3); the photoelectron backscattering amplitudes for atomic hydrogen and oxygen (SI section S7); and the photoelectron phase shifts ϕ_back_ and ϕ_abs_ (also SI section S7), we require the Fermi momentum of water and the decay widths of Br and Cl K-shell holes. As explained in SI section S4, we have determined for the Fermi momentum of water a value of *k*_F_ = 1.055 a.u., which is what we employed in the calculations underlying the results shown in the following.

For the K-shell decay widths of Br and Cl, respectively, we took the recommended values of 0.092 a.u. and 0.021 a.u. from ref 24. (The corresponding values computed using XATOM are 0.093 and 0.023 a.u., irrespective of whether the halogen atom is in the oxidation state of 0 or −1.) Note that in the range of photoelectron kinetic energies of relevance to EXAFS, the absolute value of the imaginary part of the self-energy of a photoelectron in water does not exceed about 0.08 a.u. (Figure S3). Therefore, when considering Br^–^ in water, Γ_*i*_/2 is comparable to the imaginary part of the self-energy in eq 14, whereas for Cl^–^ in water, core-hole decay is much less important than inelastic scattering of the photoelectron.

### 3.1 Impact of the Dielectric Solvent on EXAFS

The dielectric solvent enters via the complex dissipative self-energy of the photoelectron (see eqs 9–11), i.e., we expect that the presence of the dielectric solvent leads to a modification of the oscillation frequency and the decay characteristics of the pure EXAFS signal. Moreover, the role of the finite inelastic momentum transfer of the photoelectron to the solvent is of interest. We shall investigate both aspects in this subsection.

#### 3.1.1 Pure EXAFS

In this paper, we refer to as *pure* EXAFS the situation when the impact of the dielectric background is neglected. We obtain the pure EXAFS signal by setting $M\left(\sqrt{2E},E\right)=0$ in eq 14, so that, in this special case, the complex photoelectron momentum goes over into

15 $$\mathrm{k̃}\left(E\right)=\sqrt{2\left(E+i\frac{\Gamma_{i}}{2}\right)}$$

It is this *k̃* that we then insert into eq 13 for pure EXAFS, which yields basically the ordinary EXAFS equation.^5^

To illustrate the quantitative difference between the case of pure EXAFS and the case with the dielectric background, we consider the artificial, but highly well-defined situation in which only a single O atom is placed as a scatterer at a distance of 6.44 a.u. from a Br^–^ anion acting as the X-ray absorber. We then use eq 13 to compute the Br K-edge EXAFS spectrum χ­(*k*) with and without the photoelectron self-energy, i.e., with or without a homogeneous background of dynamically responsive water. [Since there is only a single atomic scatterer in the scenario considered in this subsection, only a single *n* ≠ 0 contributes to the sum in eq 13.] The comparison is shown in Figure 1. We observe that the dissipative dielectric induces a more effective dephasing of the photoelectron wave, leading to a faster decay of the EXAFS oscillations. In addition, the oscillation frequency of χ­(*k*) is altered.

**Figure 1:**
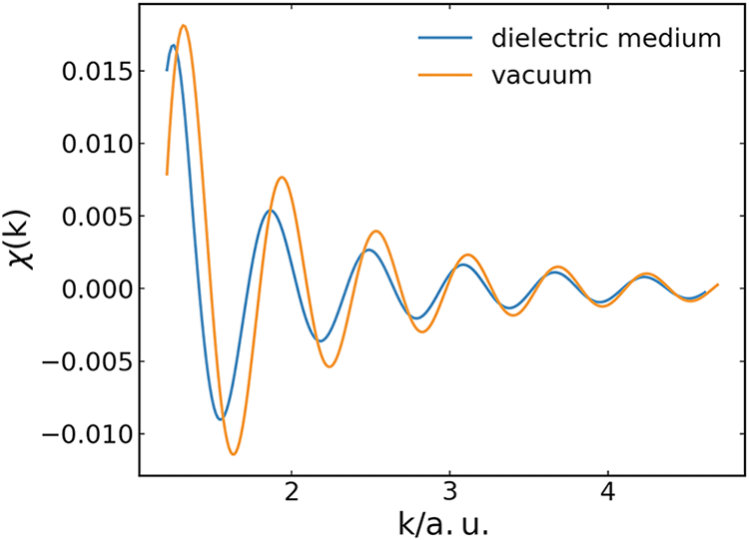
Br K-edge EXAFS spectrum χ­(*k*) for one Br^–^ anion as X-ray absorber and one O atom as scatterer placed in a homogeneous dielectric medium reflecting the dynamical response of water (blue) and in vacuum (orange). The distance between Br^–^ and O is set to 6.44 a.u.

#### 3.1.2 Role of Inelastic Momentum Transfer

Next, we investigate the impact of the *q* dependence of the dielectric function *ε*(ω, *q*) on the EXAFS signal. When computing the photoelectron self-energy (eq 10), one must integrate over all momenta that the photoelectron may transfer to the solvent, treated as a homogeneous dielectric medium. In so doing, one may either take into consideration that the dielectric function is not a constant as a function of the momentum transfer, *q*, or one may attempt to approximate *ε*(ω, *q*) by *ε*(ω, *q* = 0). The latter approximation makes it possible to reduce the numerical integration required from two dimensions to one dimension.

Again, we consider the X-ray excitation of a K-shell electron of a single Br^–^ anion solvated in water and select one O atom of a single water molecule as the photoelectron scatterer, placed at a distance of 6.44 a.u. from the X-ray absorber. Both atoms are embedded in the solvent described as a dielectric continuum. Again, we employ eq 13, i.e., only a single scattering interaction of the photoelectron with the scatterer is considered. The impact of approximating *ε*(ω, *q*) by *ε*(ω, *q* = 0), i.e., assuming that the dielectric medium has the same response at any *q* ≠ 0 as at *q* = 0, is shown in Figure 2. Clearly, taking into consideration the *q* dependence of the dielectric function weakens the decay of the EXAFS signal, but only slightly alters its oscillation frequency.

**Figure 2:**
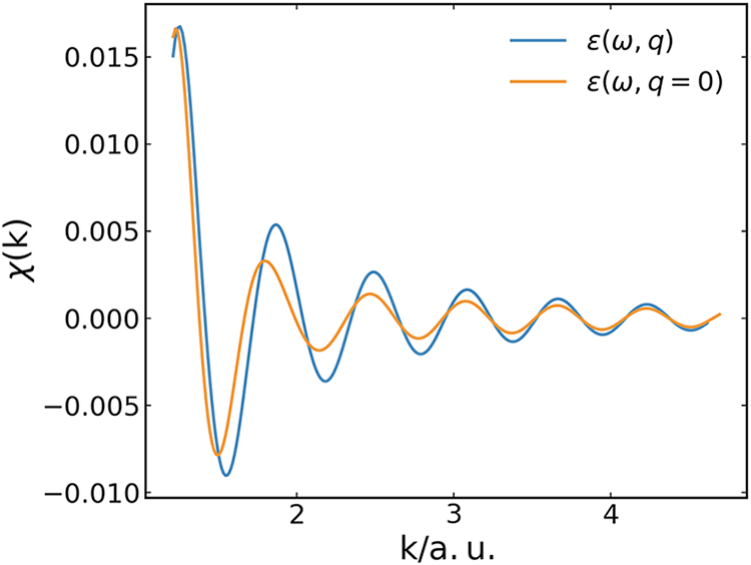
Comparison of the dissipative Br K-edge EXAFS spectrum χ­(*k*) for one Br^–^ anion and a single scattering O atom solvated in water with a *q*-dependent dielectric function of water (blue) and using the approximation *ε*(ω, *q*) = *ε*(ω, *q* = 0) (orange). The distance between the absorber Br^–^ and the scatterer O is 6.44 a.u.

### 3.2 Calculations Based on QM/MM Structures

In this subsection, we apply the framework from section 2 to structural configurations available from quantum mechanics/molecular mechanics (QM/MM) simulations^22^ and perform the configuration averaging necessary to capture the structural disorder inherently present in a liquid such as water. We demonstrate that the single-scattering approximation underlying eq 13, in combination with our specific parametrization of the dielectric function of water and with atomic parameters computed using XATOM, reproduces experimental EXAFS data quite well. In order to rationalize the origin of the success of our single-scattering approach, we utilize FEFF to determine the impact of multiple-scattering effects.

#### 3.2.1 Comparison to Experimental Data

Reference 22 provides five distinct, preequilibrated QM/MM molecular-dynamics trajectories of a Br^–^ anion embedded in an environment consisting of 199 H_2_O molecules. Thus, for any given spatial arrangement of these atoms, the index *n* ≠ 0 in eq 13 runs over 199 O atoms and 398 H atoms. Each trajectory covers a time interval of 20 ps, which we sample at a time step of 10 fs. Thus, we obtain a total of 5 × 20,000/10 = 10,000 different structural configurations. For each of these, we compute χ­(*k*) using eq 13, and then we take the ensemble average.

In Figure 3, we compare the ensemble-averaged EXAFS spectrum obtained in this way (black line) with experimental data^36^ (blue symbols) for the case of Br^–^ in water. Note that in experiment, *k* must be determined by estimating the photoelectron energy E at a given X-ray photon energy. This requires an estimate of the K-shell binding energy. Since the rise of the absorption signal at an inner-shell absorption edge is substantially broadened by core-hole decay, there is a certain degree of uncertainty regarding the exact inner-shell binding energy for a given system.^22,37^ Taking this into consideration, we have matched our theoretical data to the experimental data by applying an energy shift ΔE of +8.93 eV to our calculated, ensemble-averaged EXAFS spectrum (black line in Figure 3a). To this end, we first shifted the EXAFS spectrum in energy space by ΔE in the theoretical calculation to align it to the energy grid of the measured spectrum. Afterward, the shifted EXAFS spectrum was transformed back from energy space to wavenumber space. In passing, we note that it is a generally applied procedure to shift the absolute reference value of calculated EXAFS spectra in momentum space when they are compared to experimental data. Energy shifts up to within ±20 eV are common in the literature.^37^ The general shape of EXAFS spectra is not affected by this shift. The physical mechanisms at the origin of the shift are not included in the theoretical calculation of EXAFS spectra. Given the substantial number of approximations made in this work, the agreement with the experimental data is satisfactory.

**Figure 3:**
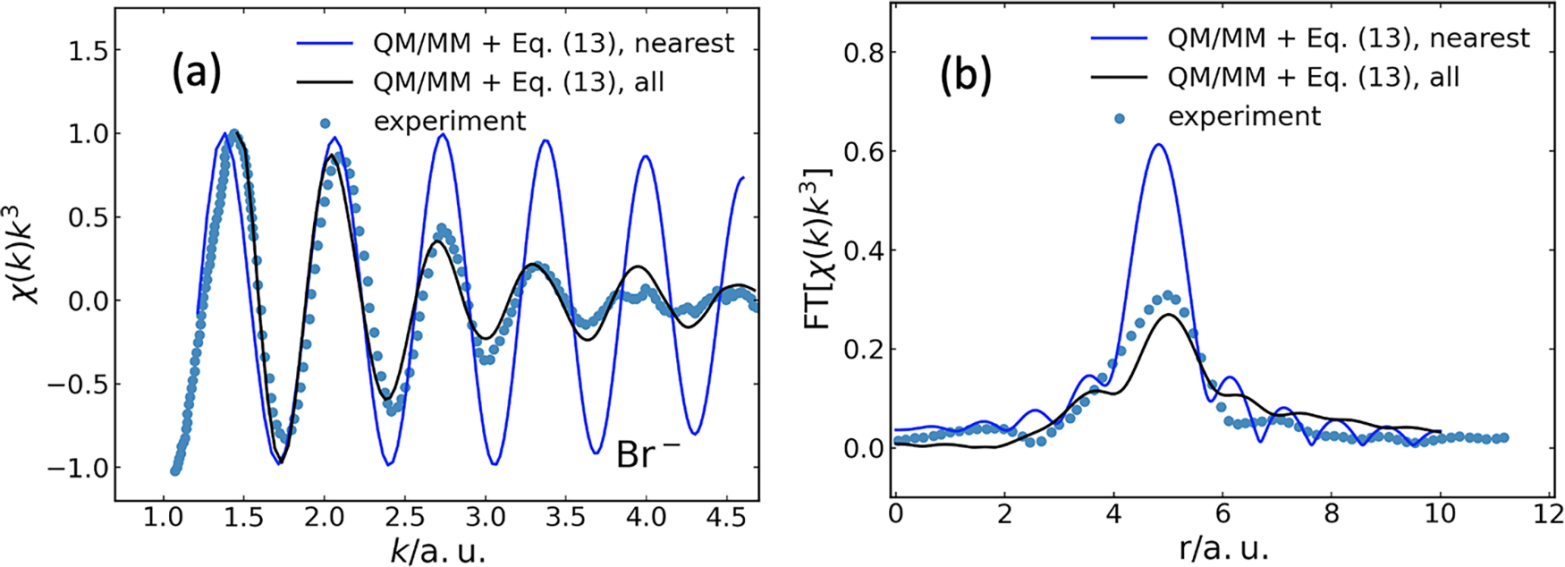
(a) Br K-edge EXAFS spectrum χ­(*k*)*k* ^3^ as a function of the photoelectron wavenumber *k* for Br^–^ dissolved in water. The black line marks the result obtained using eq 13, after averaging over 10,000 QM/MM equilibrium configurations, as described in the text. The blue symbols mark the experimental spectrum from ref 36. The blue line indicates the calculated EXAFS spectrum when including, in addition to the absorber, only the O atom nearest to the absorber in each QM/MM configuration. Also in this case, the average over the ensemble of QM/MM configurations was taken. The spectra are normalized to their individual maxima. (b) The corresponding Fourier transform of the EXAFS spectrum to real space (without phase-shift correction). Experimental data are reproduced with permission from ref 36. Copyright 2016 AIP Publishing.

In Figure 3b, we show the corresponding Fourier transforms of χ­(*k*)*k* ^3^ to real space. We find that the maxima of the signals, which indicate the average of the atomic position of the dominant scatterer, almost coincide. The calculated ensemble-averaged EXAFS spectrum shows three local maxima. The largest peak corresponds to the dominating O atom, while the two smaller peaks to the left and the right correspond to the two associated H atoms. The experimental data are not able to resolve these separate peaks.

Also shown in Figure 3 is the situation in which we restricted photoelectron backscattering to the oxygen atom closest to the photoelectron parent atom; the other 198 oxygen atoms, and all hydrogen atoms, in each QM/MM configuration were excluded from the sum over *n* ≠ 0 in eq 13. The resulting EXAFS spectrum, obtained after averaging over the 10,000 QM/MM snapshots considered, is shown as the blue line in Figure 3a. Apparently, this EXAFS spectrum matches neither the experimental data nor the spectrum calculated with all water molecules in the environment of the X-ray absorber. It decays much more slowly, in the regime considered, than the other two spectra, albeit the oscillation frequencies match well. This translates to real space via the Fourier transforms, as shown in Figure 3b: There is similar structural information in all three cases. Yet, the remaining atoms in the environment are essential to capture the decay of the EXAFS signal with increasing *k*.

#### 3.2.2 Role of Multiple-Scattering Effects

We next assess the role of multiple-scattering events. For this, we utilize FEFF to calculate EXAFS spectra, employing the QM/MM configurations from ref 22. as input data for FEFF. The results obtained upon averaging over the QM/MM ensemble are shown in Figure 4. Since we take structural fluctuations into consideration through the QM/MM ensemble used, the Debye–Waller factor was set to 1 in our FEFF calculations. Apart from that, we used default FEFF parameters. Particularly, we did not employ the many-pole self-energy introduced in ref 23., but used only the standard treatment of dissipation in FEFF. For the single-scattering calculations, we set the FEFF parameter NLEG to 2.

**Figure 4:**
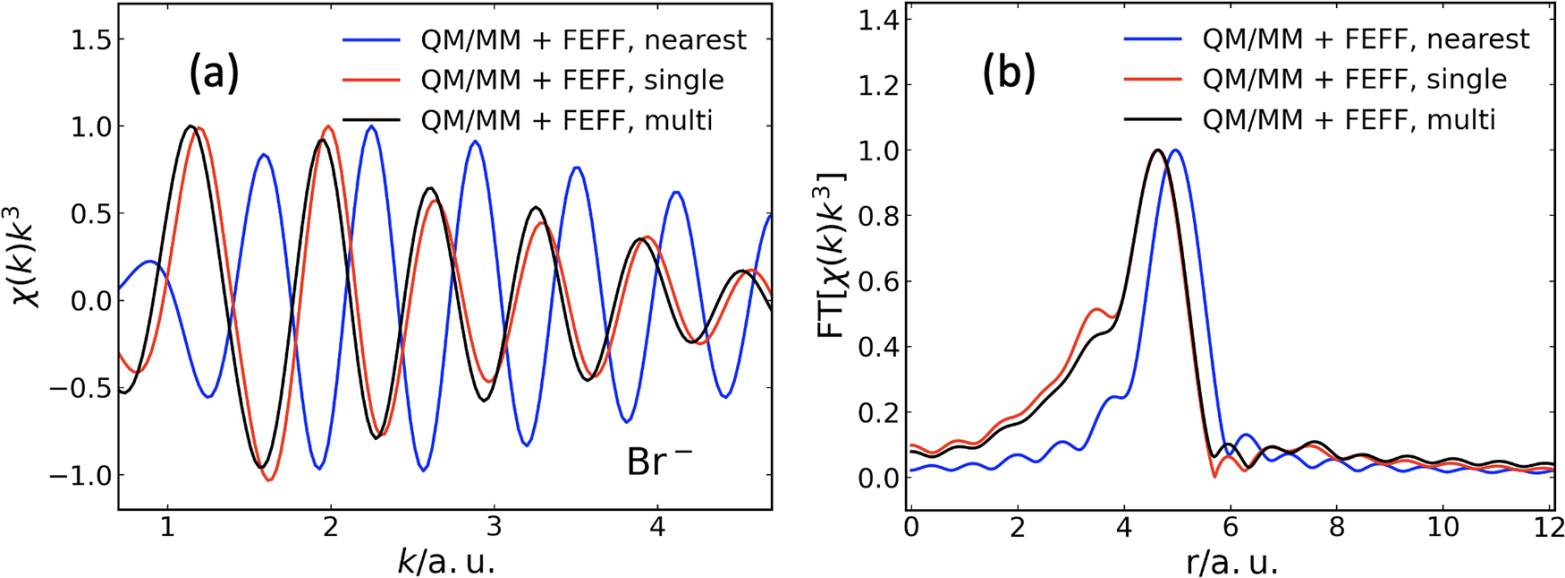
(a) Ensemble-averaged Br K-edge EXAFS spectrum χ­(*k*)*k* ^3^, calculated using FEFF, for a Br^–^ absorber embedded in water. The underlying structural configurations are taken from QM/MM simulations.^22^ No energy shift is applied, and all spectra are normalized to their respective maximum. Blue: only the nearest oxygen atom is considered as scatterer. Red: single scattering. Black: multiple scattering. (b) Corresponding Fourier transform (without phase-shift correction).

In Figure 4, we consider three different constellations. First, we include, in addition to the absorber atom, only the nearest oxygen atom in each QM/MM configuration. The result is shown in blue in Figure 4. Second, in red in Figure 4 we display the EXAFS spectrum computed at the single-scattering level of theory. Third, the result obtained by using the multiple-scattering capability of FEFF is depicted in black in Figure 4.

By comparing the red and black lines in Figure 4, we may conclude that multiple-scattering effects contribute to slight modifications of the decay and the phase shift of the oscillations, but do not cause a significant change in frequency. This is also reproduced in the Fourier transforms, which peak at the same position. In particular, we can confirm the conclusion already drawn in section 3.2.1: The main contribution to the EXAFS spectrum comes from the single-scattering pathways. Multiple-scattering effects seem less important. This is ultimately a consequence of the structural fluctuations characterizing a liquid such as water. These fluctuations cause the contributions from multiple scattering to wash out.

This is further illustrated through Figure 5, where we compare single-scattering and multiple-scattering FEFF calculations for five of the 10,000 QM/MM configurations underlying Figure 4. As may be seen in Figure 5, for individual structural configurations the impact of multiple scattering is more evident than is the case for the ensemble average shown in Figure 4. Nevertheless, there is more variation among the spectra for different structural configurations than is the case between single- and multiple-scattering spectra for a given structural configuration. This means that even in individual structural configurations representing microscopic snapshots of a liquid-phase medium, there is enough disorder to cause a suppression of photoelectron scattering contributions beyond single scattering. Therefore, Figures 4 and 5 suggest that the theoretical framework presented in section 2 could generally be useful for computing EXAFS spectra of liquid-phase systems, not only for the hydrated halides considered in this work.

**Figure 5:**
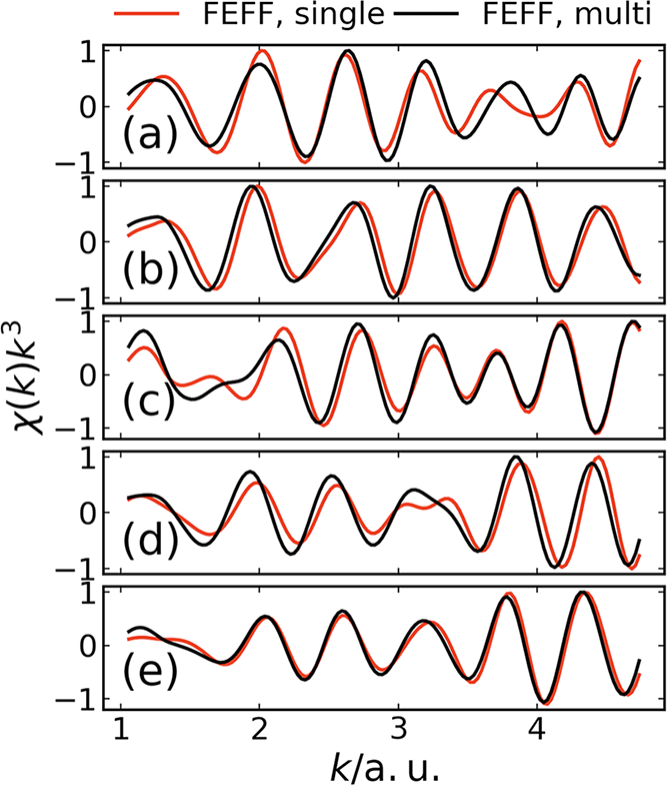
(a–e) Br K-edge EXAFS spectra χ­(*k*)*k* ^3^, calculated using FEFF, for five randomly selected structural configurations from ref 22. No energy shift is applied, and all spectra are normalized to their respective maximum. Red: single scattering. Black: multiple scattering.

#### 3.2.3 Hydrated Chloride

In addition to the case of solvated Br^–^, we apply the framework presented in section 2 also to solvated Cl^–^ in water. To calculate the EXAFS spectrum of Cl^–^ solvated in water, we again utilize eq 13, employing 10,000 structural configurations determined by QM/MM simulations,^22^ following the same protocol as before. In this case, however, no energy shift is applied. The computed EXAFS spectrum, averaged over all 10,000 QM/MM configurations, is compared in Figure 6 with experimental data. Again, given the simplicity of our model, the agreement with experiment is quite good.

**Figure 6:**
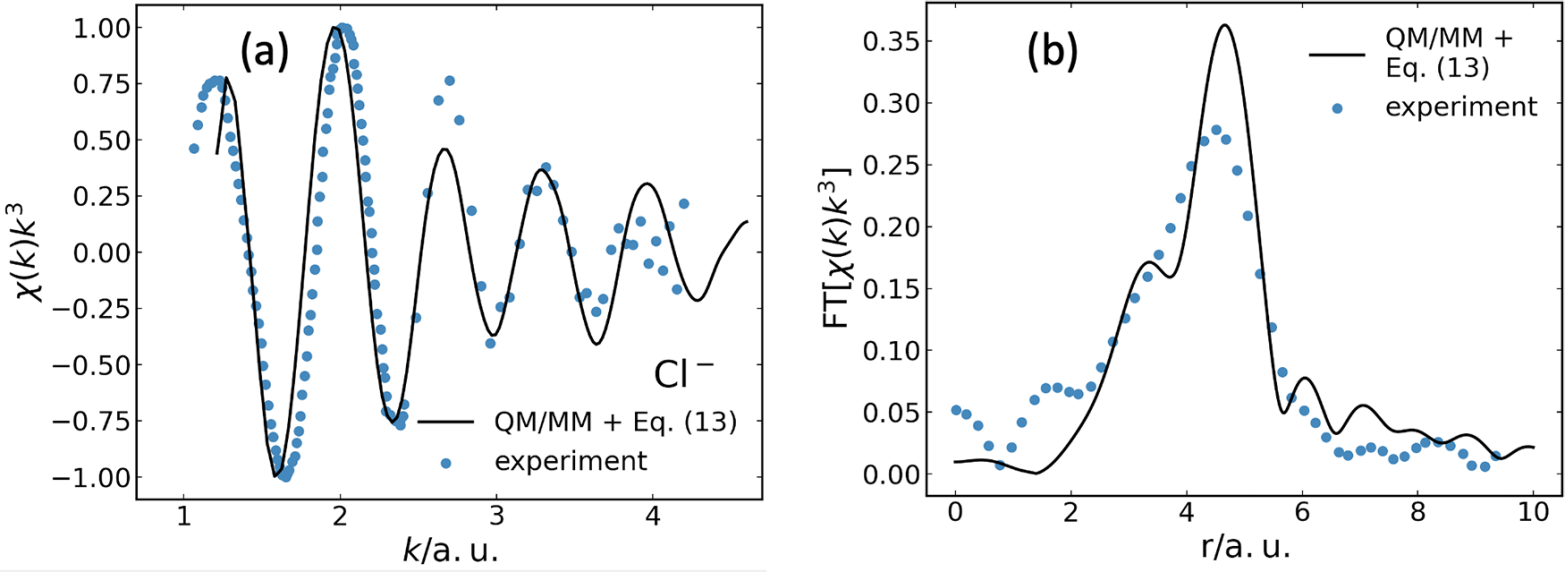
(a) Ensemble-averaged Cl K-edge EXAFS spectrum χ­(*k*)*k* ^3^, as a function of the photoelectron wavenumber, for Cl^–^ in water and (b) corresponding Fourier transform. Black lines show the result obtained using eq 13, after averaging over 10,000 QM/MM equilibrium configurations. Blue symbols mark experimental data from ref 36. The EXAFS spectra are normalized to their respective maximum. No energy shift is used. Experimental data are reproduced with permission from ref 36. Copyright 2016 AIP Publishing.

## 4 Conclusions

When single atoms or molecules are dissolved in a liquid solvent, the disordered nature of the material hosting the photoelectron still poses challenges for an accurate calculation of the EXAFS spectrum. We have shown in this work that an independent-atom single-scattering description in terms of the photoelectron’s Green’s function in the GW approximation is a viable approach to atomistic simulations when the host solvent is described by an accurately parametrized complex dielectric function. The dephasing and relaxation of the photoelectron occurs due to inelastic transfer of energy and momentum to the solvent. It enters via the complex dielectric function in the complex self-energy of the photoelectron and results in a frequency shift of the EXAFS signal as compared to the vacuum, and in an enhanced decay. Clearly, the optical regime of the dielectric function is relevant at these ultrafast sub-1 fs time scales.

We validated the proposed approach through calculation of the inelastic mean free path (IMFP) of the photoelectron in water (SI section S5), for which theoretical results are available in the literature, and of the EXAFS spectra of single bromide and chloride anions dissolved in water, for which a comparison with experimental data is possible. A conceptually interesting conclusion of our work is that, in liquid-phase media, the role of multiple-scattering events is secondary as compared to the role of the dielectric solvent and the inelastic energy and momentum exchange. In general, the single-scattering approximation can only be rigorously justified in the high-energy limit, i.e., in the energy range required for the validity of the first Born approximation. However, it is well-known^38^ that, generally, it is important to take multiple-scattering pathways into consideration in the photoelectron energy range of relevance for EXAFS signals. In other words, for general chemical environments, it is by no means obvious that the single-scattering approximation may be expected to work. Our results demonstrate that for water, i.e., the universally most important solvent, the single-scattering approximation to EXAFS appears to be quantitatively accurate. Whether this conclusion is specific to halogen anions or whether it applies, as we suspect, also to other solutes in water, will require research beyond the scope of this paper.

It might be argued that for more structured liquids, or for higher-Z scatterers, multiple scattering may be enhanced. For locally more structured liquids, the structure is still not long-range periodic. Therefore, the contributions from multiple-scattering paths will still be damped as a consequence of disorder. Hence, it may be expected that the single-scattering approximation remains mostly valid in more structured liquids, but including key multiple-scattering paths can improve accuracy if strong local geometry correlations exist. Similarly, in liquids, even with heavier scatterers, the atomic arrangement is highly disordered. As a consequence, the contributions from multiple-scattering paths have a tendency to average out. In this sense, it is likely that our method remains valid for more structured liquids, or in the presence of higher-Z scatterers. But more research is needed to clarify to what degree the single-scattering approximation is useful in such situations.

Our results suggest that when structural configurations from molecular-dynamics calculations are available, in addition to a sufficiently accurate dielectric function (either measured or calculated), then the independent-atom single-scattering approach presented in this work permits the prediction of EXAFS spectra at relatively low computational cost. All atomic parameters required may be precomputed using XATOM. The QM/MM simulations reported in the present work took approximately 72 CPUh and the subsequent EXAFS calculation took about 2 CPUh on a standard contemporary local computer. Pushing such calculations to much larger and more complex systems is already, to some degree, routine. Pure MM-based classical molecular dynamics is nowadays quite standard for systems consisting of 105 atoms or more (see, e.g., ref 39). The real bottleneck is the quantum mechanical (QM) part. But even pure QM-based molecular dynamics (known as “ab initio molecular dynamics” or AIMD) has already been extended to similarly large systems (see, e.g., ref 40). Recent examples from the literature^41–43^ illustrate that QM/MM methodology is sufficiently advanced to tackle molecular systems of significantly higher levels of complexity than those considered in our present work.

## Supplementary Material



## Notes

Details on the theoretical methods summarized in section 2: derivation of the inner-shell photoabsorption cross section (section S1); retarded one-particle Green’s function and self-energy (section S2); parametrization of the dielectric function of water (section S3); evaluation of the dissipative photoelectron self-energy (section S4); validation of the dissipative photoelectron self-energy (section S5); evaluation of the absorption cross section (section S6); determination of the relative EXAFS signal (section S7) (PDF)
