# Supplementary material for: Effective Quantum Theory of EXAFS in a Dissipative Liquid-Phase Medium
Source: J Phys Chem B. 2025 Dec 18;130(1):374–83. doi: 10.1021/acs.jpcb.5c06230 (PMC12794161; doi:10.1021/acs.jpcb.5c06230)
Supplement: Supplementary file 1 [file jp5c06230_si_001.pdf]

Effective Quantum Theory of EXAFS in a Dissipative Liquid-phase Medium: Supporting Information

Mei Bai,[†,‡] Robin Santra,[¶,†,‡] Sang-Kil Son,[¶,‡] Henning Kirchberg,[§] and Michael Thorwart[*,†,‡]

† *I. Institut für Theoretische Physik, Universität Hamburg, Notkestraße 9, 22607 Hamburg, Germany*

‡ *The Hamburg Centre for Ultrafast Imaging, Universität Hamburg, Luruper Chaussee 149, 22761 Hamburg, Germany*

¶ *Center for Free-Electron Laser Science CFEL, Deutsches Elektronen-Synchrotron DESY, Notkestr. 85, 22607 Hamburg, Germany*

§ *Department of Microtechnology and Nanoscience, Chalmers University of Technology, Kemivägen 9, 41296 Göteborg, Sweden*

E-mail: michael.thorwart@uni-hamburg.de

S1 Derivation of the Inner-shell Photoabsorption Cross Section

S1.1 Conceptual Foundations

We build on the following key assumptions:[S1]

1. The absorbing atomic species consists of individual atoms that are surrounded by other atomic species forming the solvent. Under this condition, we can place a representative of the absorbing species at the origin of our coordinate system and use the electric-dipole approximation.

2. For x-ray absorption by true inner shells, there is normally only little interchannel coupling. This means that, to a good approximation, we can treat the hole and the particle in the particle-hole-excited state as two independent quasiparticles.

3. The x-ray beam is assumed to be sufficiently monochromatized so that spectral-band-width effects may be neglected. For convenience, we employ a single-mode Fock state for the incoming x-ray field.

4. Neither the photoelectron nor the hole is detected. This means that the signal of interest involves a sum over all accessible particle-hole states. All that matters is the photoabsorption rate.

5. For a full quantum treatment, including a quantum-mechanical description of the transverse part of the electromagnetic field, it is convenient to employ the technique of adiabatic switching.

We employ the framework of nonrelativistic quantum electrodynamics. Hence, our starting point is the full Hamiltonian[S1]

$$\hat{H} = \hat{H}_0 + \hat{H}_{\mathrm{int}}, \tag{S1}$$

with the noninteracting Hamiltonian

$$\hat{H}_0 = \hat{H}_{\mathrm{matter}} + \hat{H}_{\mathrm{EM}}, \tag{S2}$$

and with the Hamiltonian of the matter part in the form (in atomic units)

$$\begin{aligned}\hat{H}_{\mathrm{matter}} &= \int d^3x \hat{\psi}^\dagger(\vec{x}) \left\{ -\frac{1}{2}\vec{\nabla}^2 - \sum_n \frac{Z_n}{|\vec{x}-\vec{R}_n|} \right\} \hat{\psi}(\vec{x}) \\ &+ \frac{1}{2}\int d^3x \int d^3x' \hat{\psi}^\dagger(\vec{x})\hat{\psi}^\dagger(\vec{x}')\frac{1}{|\vec{x}-\vec{x}'|}\hat{\psi}(\vec{x}')\hat{\psi}(\vec{x}) + V_{\mathrm{N-N}}\,. \end{aligned} \tag{S3}$$

Here, $\hat{\psi}(\vec{x})$ is the electronic field operator, which is a two-component spinor,

$$\hat{\psi}(\vec{x}) = \begin{pmatrix} \hat{\psi}_{+1/2}(\vec{x}) \\ \hat{\psi}_{-1/2}(\vec{x}) \end{pmatrix}, \tag{S4}$$

$\vec{R}_n$ is the position of the n-th nucleus, Z_n the nuclear charge, and $V_{\mathrm{N-N}}$ represents the nucleus-nucleus interaction.

The electromagnetic-field Hamiltonian is given as

$$\hat{H}_{\mathrm{EM}} = \sum_{\vec{k},\lambda} \omega_{\vec{k}} \hat{a}^\dagger_{\vec{k},\lambda} \hat{a}_{\vec{k},\lambda}\,, \tag{S5}$$

with the electromagnetic-field mode operators $\hat{a}_{\vec{k},\lambda}$ for the photon wave vector $\vec{k}$ and the polarization index $\lambda = 1, 2$. The photon frequency in atomic units is $\omega_{\vec{k}} = k/\alpha$ with $k = |\vec{k}|$, α being the fine-structure constant. The associated vector potential is

$$\hat{\vec{A}}(\vec{x}) = \sum_{\vec{k},\lambda} \sqrt{\frac{2\pi}{V\omega_{\vec{k}}\alpha^2}} \{ \hat{a}_{\vec{k},\lambda} \epsilon_{\vec{k},\lambda} e^{i\vec{k}\cdot\vec{x}} + \hat{a}^\dagger_{\vec{k},\lambda} \epsilon^*_{\vec{k},\lambda} e^{-i\vec{k}\cdot\vec{x}} \} \tag{S6}$$

with the polarization vector $\epsilon_{\vec{k},\lambda}$ and the volume V of a box as a reference with respect to which the plane-wave states are normalized. The interaction Hamiltonian is given in the

electric-dipole approximation by

$$\hat{H}_{\text{int}} = \alpha \int d^3x \hat{\psi}^\dagger(\vec{x}) \frac{\vec{\nabla}}{i} \cdot \hat{\vec{A}}(\vec{0}) \hat{\psi}(\vec{x}) \,. \tag{S7}$$

S1.2 Absorption Cross Section and Extended X-ray Absorption Fine Structure (EXAFS) Signal

S1.2.1 Formally Exact Expressions

To determine the absorption cross section, we proceed with the Schrödinger equation in the interaction picture

$$i \frac{\partial}{\partial t} \left|\Psi(t)\right\rangle_{\text{int}} = \hat{H}_{\text{int}}(t) \left|\Psi(t)\right\rangle_{\text{int}} \,, \tag{S8}$$

with the interaction Hamiltonian in the interaction picture as

$$\hat{H}_{\text{int}}(t) = e^{-\zeta|t|} e^{i\hat{H}_0 t} \hat{H}_{\text{int}} e^{-i\hat{H}_0 t} \,. \tag{S9}$$

Its iterative formal solution

$$\left|\Psi(t)\right\rangle_{\text{int}} = \left|I\right\rangle - i \int_{-\infty}^{t} dt_1 \hat{H}_{\text{int}}(t_1) \left|I\right\rangle - \int_{-\infty}^{t} dt_2 \hat{H}_{\text{int}}(t_2) \int_{-\infty}^{t_2} dt_1 \hat{H}_{\text{int}}(t_1) \left|I\right\rangle + \ldots \tag{S10}$$

contains the initial state $|I\rangle$, which will be specified below.

The probability amplitude in the interaction picture to remain in the initial state is given by[S2]

$$\beta_I(t) = \left\langle I | \Psi(t) \right\rangle_{\text{int}} = 1 - \int_{-\infty}^{t} dt_2 \int_{-\infty}^{t_2} dt_1 \left\langle I | \hat{H}_{\text{int}}(t_2) \hat{H}_{\text{int}}(t_1) | I \right\rangle \,. \tag{S11}$$

With the initial-state probability $P_I(t) = \beta_I^*(t)\beta_I(t)$, the total absorption rate follows as

$$\Gamma_{\text{abs}} = -\dot{P}_I(t) = -\{\dot{\beta}_I^*(t)\beta_I(t) + \beta_I^*(t)\dot{\beta}_I(t)\} \simeq -\{\dot{\beta}_I^*(t) + \dot{\beta}_I(t)\} = -2\,\text{Re}\{\dot{\beta}_I(t)\} \,, \tag{S12}$$

where we have used that $\beta_I(t) \simeq 1$. Using Eq. (S11), we find

$$\begin{aligned}\Gamma_{\text{abs}} &= 2\,\text{Re}\left\{\int_{-\infty}^{t} dt_1\, \langle I|\hat{H}_{\text{int}}(t)\hat{H}_{\text{int}}(t_1)|I\rangle\right\} \\ &= 2\,\text{Re}\left\{\int_{-\infty}^{t} dt_1\, \langle I|\hat{H}_{\text{int}} e^{-i(\hat{H}_0 - E_I)(t-t_1)} \hat{H}_{\text{int}}|I\rangle\, e^{-\zeta|t_1|}\right\} \\ &= 2\,\text{Im}\left\{\langle I|\hat{H}_{\text{int}} \frac{1}{\hat{H}_0 - E_I - i\zeta} \hat{H}_{\text{int}}|I\rangle\right\}. \end{aligned} \tag{S13}$$

Next, we specify the initial state as

$$|I\rangle = |\Psi_0\rangle\, |N_x\rangle \tag{S14}$$

as a product state of the electronic initial state $|\Psi_0\rangle$, which is typically the ground state, and of the photonic Fock state $|N_x\rangle$ with $N_x = \langle N_x| \sum_{\vec{k},\lambda} \hat{a}^\dagger_{\vec{k},\lambda} \hat{a}_{\vec{k},\lambda} |N_x\rangle$ x-ray photons. Upon specifying $\hat{H}_0$ and $\hat{H}_{\text{int}}$, we obtain the photoabsorption rate in the explicit form as

$$\begin{aligned}\Gamma_{\text{abs}} &= \frac{4\pi}{\omega_x}\frac{N_x}{V} \int d^3x \int d^3x' \qquad \text{(S15)} \\ &\quad \times \text{Im}\left\{\langle \Psi_0|\hat{\psi}^\dagger(\vec{x}) \frac{\vec{\nabla}}{i} \cdot \vec{\epsilon}^{\,*}\ \hat{\psi}(\vec{x}) \frac{1}{\hat{H}_{\text{matter}} - E_0 - \omega_x - i\zeta} \hat{\psi}^\dagger(\vec{x}') \frac{\vec{\nabla}'}{i} \cdot \vec{\epsilon}\ \hat{\psi}(\vec{x}')|\Psi_0\rangle\right\}. \end{aligned}$$

Here, E_0 is the energy associated with the electronic eigenstate $|\Psi_0\rangle$, ω_x is the energy of the x-ray photon, and $\vec{\epsilon}$ is the x-ray polarization vector. Here, we assume that the x-ray photon is in a well-defined polarization state with a given frequency and thus wave vector. Eventually, the x-ray absorption cross section then follows as

$$\sigma_{\text{abs}} = \frac{4\pi\alpha}{\omega_x} \text{Im}\left\{\langle \Psi_0|\hat{\vec{D}} \cdot \vec{\epsilon}^{\,*}\ \frac{1}{\hat{H}_{\text{matter}} - E_0 - \omega_x - i\zeta} \hat{\vec{D}} \cdot \vec{\epsilon}\ |\Psi_0\rangle\right\}, \tag{S16}$$

where

$$\hat{\vec{D}} = \int d^3x \hat{\psi}^\dagger(\vec{x}) \frac{\vec{\nabla}}{i} \hat{\psi}(\vec{x}) \tag{S17}$$

is the electric-dipole operator in momentum form.

S1.2.2 Approximations for Inner-shell Photoionization

Apart from the electric-dipole approximation and the assumption of x-ray monochromaticity (and the assumption that the sample is electronically cold), the expression for the x-ray absorption cross section σ_{abs} in Eq. (S16) is formally exact. One might naively attempt to evaluate it by inserting a complete set of many-electron eigenstates of $\hat{H}_{\text{matter}}$, which we know no better than we know the true $|\Psi_0\rangle$.

Therefore, we next introduce assumptions more specific to the inner-shell photoionization problem. By introducing some suitable mean-field such as a restricted Hartree-Fock description of the initial state, we can expand the field operator $\hat{\psi}(\vec{x}')$ that acts on $|\Psi_0\rangle$ in terms of the spin orbitals of that mean-field. We may proceed analogously for $\langle\Psi_0|\,\hat{\psi}^\dagger(\vec{x})$. As such, this is not an approximation, but a choice.

It becomes an approximation when we assume

(a) that in the photon-energy range of interest, only one specific (spatial) inner-shell orbital i determines (dominates) the absorption cross section (e.g., the K shell of the primarily absorbing species), and,

(b) that the photoelectron is unable to change the state of the hole.

Hence, we obtain from Eqs. (S16) and (S17):

$$\begin{aligned}\sigma_{\text{abs}} &= \frac{4\pi\alpha}{\omega_x}\int d^3x \int d^3x' \sum_\sigma \operatorname{Im}\left\{\varphi^\dagger_{i\sigma}(\vec{x})\frac{\vec{\nabla}}{i}\cdot\vec{\epsilon}^{\,*}\right. \\ &\quad \left.\times\,\langle\Psi_0|\hat{c}^\dagger_{i\sigma}\hat{\psi}(\vec{x})\frac{1}{\hat{H}_{\text{matter}}-E_0-\omega_x-i\zeta}\hat{\psi}^\dagger(\vec{x}')\hat{c}_{i\sigma}|\Psi_0\rangle\,\frac{\vec{\nabla}'}{i}\cdot\vec{\epsilon}\,\varphi_{i\sigma}(\vec{x}')\right\}.\end{aligned} \tag{S18}$$

Here, we have introduced the two-component spinor

$$\varphi_{i\sigma}(\vec{x}) = \varphi_i(\vec{x})\begin{pmatrix}\delta_{\sigma,+1/2}\\ \delta_{\sigma,-1/2}\end{pmatrix}, \tag{S19}$$

with the spatial orbital $\varphi_i(\vec{x})$ and with the fermionic annihilation operator $\hat{c}_{i\sigma}$ for the inner-shell electron with spin σ.

The remaining electronic field operators may be expanded in plane waves, which yields

$$\begin{aligned}\sigma_{\text{abs}} &= \frac{4\pi\alpha}{\omega_x}\sum_\sigma \int d^3k \int d^3k' \\ &\times \text{Im}\left\{ \langle\Psi_0|\hat{c}^\dagger_{i\sigma}\hat{c}_{\vec{k}\sigma}\frac{1}{\hat{H}_{\text{matter}} - E_0 - \omega_x - i\zeta}\hat{c}^\dagger_{\vec{k}'\sigma}\hat{c}_{i\sigma}|\Psi_0\rangle\,(\vec{k}\cdot\vec{\epsilon}^{\,*})\left\langle\vec{k}\middle|i\right\rangle^*(\vec{k}'\cdot\vec{\epsilon}\,)\left\langle\vec{k}'\middle|i\right\rangle\right\}.\end{aligned} \tag{S20}$$

Here, the fermionic creation operator of the photoelectron in the plane-wave state $(\vec{k}', \sigma)$ is given by $\hat{c}^\dagger_{\vec{k}'\sigma}$, and

$$\left\langle\vec{k}\middle|i\right\rangle = \frac{1}{(2\pi)^{3/2}}\int d^3x\, e^{-i\vec{k}\cdot\vec{x}}\varphi_i(\vec{x}) \tag{S21}$$

is the overlap of the core orbital $\varphi_i(\vec{x})$ with the plane-wave photoelectron state with momentum $\vec{k}$. Equations (S20) and (S21) equal Eqs. (1) and (2) in the main text.

S2 Retarded One-particle Green's Function and Self-energy

All the remaining many-body effects in σ_{abs} are encapsulated in the function

$$\langle\Psi_0|\hat{c}^\dagger_{i\sigma}\hat{c}_{\vec{k}\sigma}\frac{1}{\hat{H}_{\text{matter}} - E_0 - \omega_x - i\zeta}\hat{c}^\dagger_{\vec{k}'\sigma}\hat{c}_{i\sigma}|\Psi_0\rangle\,, \tag{S22}$$

appearing in Eq. (S20). Because microscopically, the system is not translationally invariant, this function may not be assumed to be diagonal in $\vec{k}$. In general, one cannot guarantee that $\hat{c}_{i\sigma}|\Psi_0\rangle$ is normalized ($|\Psi_0\rangle$ is assumed to be normalized). Yet, this is different for inner shells: Unless the initial state $|\Psi_0\rangle$ is inner-shell excited, electron-electron interactions are not strong enough to cause a significant admixture from inner-shell-excited configurations. Hence, we may safely assume that

$$\hat{c}^\dagger_{i\sigma}|\Psi_0\rangle = 0\,, \tag{S23}$$

which implies

$$\langle\Psi_0|\hat{c}^\dagger_{i\sigma}\hat{c}_{i\sigma}|\Psi_0\rangle = \langle\Psi_0|(1-\hat{c}_{i\sigma}\hat{c}^\dagger_{i\sigma})|\Psi_0\rangle = \langle\Psi_0|\Psi_0\rangle = 1\,. \tag{S24}$$

Thus, it is tempting to identify the object

$$\begin{aligned}
&\langle\Psi_0|\hat{c}^\dagger_{i\sigma}\hat{c}_{\vec{k}\sigma}\frac{1}{\hat{H}_{\text{matter}}-E_0-\omega_x-i\zeta}\hat{c}^\dagger_{\vec{k}'\sigma}\hat{c}_{i\sigma}|\Psi_0\rangle \\
=\;& \langle\Psi_0|\hat{c}^\dagger_{i\sigma}\hat{c}_{\vec{k}\sigma}\frac{1}{\hat{H}_{\text{matter}}-E_{i\sigma}-(\omega_x-[E_{i\sigma}-E_0])-i\zeta}\hat{c}^\dagger_{\vec{k}'\sigma}\hat{c}_{i\sigma}|\Psi_0\rangle \\
=\;& \langle\Psi_0|\hat{c}^\dagger_{i\sigma}\hat{c}_{\vec{k}\sigma}\frac{1}{\hat{H}_{\text{matter}}-E_{i\sigma}-(\omega_x-I_i)-i\zeta}\hat{c}^\dagger_{\vec{k}'\sigma}\hat{c}_{i\sigma}|\Psi_0\rangle
\end{aligned} \tag{S25}$$

with the inner-shell ionization potential

$$I_i = E_{i\sigma} - E_0 \tag{S26}$$

as a retarded one-particle Green's function[S3] for the state $\hat{c}_{i\sigma}\,|\Psi_0\rangle$ (up to a sign). What limits this interpretation is that $\hat{c}_{i\sigma}\,|\Psi_0\rangle$ may not, strictly speaking, be viewed as an eigenstate of $\hat{H}_{\text{matter}}$. On the other hand, we recognize that the interpretation of EXAFS rests on the assumption that shake-up and shake-off satellites may be neglected. For this reason, we treat the state

$$|\Psi_{i\sigma}\rangle \equiv \hat{c}_{i\sigma}\,|\Psi_0\rangle \tag{S27}$$

as if it corresponded to the ionization channel associated with the photoelectron main line, with the ionization potential I_i for the inner-shell orbital i and with the decay rate Γ_i of the inner-shell hole state.

Moreover, the corresponding advanced Green's function

$$\langle\Psi_0|\hat{c}^\dagger_{i\sigma}\hat{c}^\dagger_{\vec{k}'\sigma}\frac{1}{E_{i,\sigma}-\hat{H}_{\text{matter}}-(\omega_x-I_i)+i\zeta}\hat{c}_{\vec{k}\sigma}\hat{c}_{i\sigma}|\Psi_0\rangle \tag{S28}$$

is significantly off-shell. Hence, for our purposes, it makes no practical difference to replace the retarded Green's function with the time-ordered one. This step brings the advantage

that we can directly use the Dyson equation and write the one-particle Green's function in terms of a self-energy.[S3]

A possible approach is to perform individual atomic-structure calculations with a local exchange potential for the atoms in the system. We implement this strategy using XATOM (see Sec. S7). In this way, we obtain a local scattering potential

$$V_{\mathrm{sc}}(\vec{x}) = \sum_n V_n(|\vec{x} - \vec{R}_n|)\,, \tag{S29}$$

where n runs over all atoms in the system. We will assume that $n = 0$ corresponds to the parent atom of the photoelectron. This approach implies that $V_{\mathrm{sc}}(\vec{x})$ includes an approximation to both the direct and the exchange interaction of the photoelectron with the other electrons. It also includes an approximation to the interaction of the photoelectron with all nuclei. $\hat{H}_{\mathrm{matter}}$ would then be written, for the purpose of many-body perturbation theory, as a sum of the unperturbed part

$$\int d^3x\, \hat{\psi}^\dagger(\vec{x}) \left\{-\frac{1}{2}\vec{\nabla}^2 + V_{\mathrm{sc}}(\vec{x})\right\} \hat{\psi}(\vec{x}) \tag{S30}$$

(the nucleus-nucleus interaction is not relevant here and is, therefore, dropped), and the perturbation

$$\begin{aligned} &\frac{1}{2}\int d^3x \int d^3x'\, \hat{\psi}^\dagger(\vec{x})\hat{\psi}^\dagger(\vec{x}')\frac{1}{|\vec{x}-\vec{x}'|}\hat{\psi}(\vec{x}')\hat{\psi}(\vec{x}) \\ &-\sum_n \int d^3x\, \hat{\psi}^\dagger(\vec{x}) \left\{V_n(|\vec{x}-\vec{R}_n|) + \frac{Z_n}{|\vec{x}-\vec{R}_n|}\right\} \hat{\psi}(\vec{x})\,. \end{aligned} \tag{S31}$$

This means that the self-energy that appears in the interacting Green's function depends on what precisely is used to define the noninteracting Green's function in the Dyson equation. Particularly, since the exchange interaction is incorporated to some degree in $V_{\mathrm{sc}}(\vec{x})$, we will omit it from the self-energy, because otherwise there would be a certain degree of double counting. See Sec. S4 for more details.

We implement the described reasoning by replacing the retarded one-particle Green's function in Eq. (S25) by

$$-G_{\vec{k}\vec{k}'}(\mathcal{E}=\omega_x-I_i)=\left\langle\vec{k}\middle|\frac{1}{-\frac{1}{2}\vec{\nabla}^2+V_{\text{sc}}+\mathcal{M}(\mathcal{E}=\omega_x-I_i)-i\frac{\Gamma_i}{2}-(\omega_x-I_i)}\middle|\vec{k}'\right\rangle\,. \tag{S32}$$

In this expression, which equals Eq. (3) in the main text, $\mathcal{M}(\mathcal{E}=\omega_x-I_i)$ is the photoelectron self-energy that results from the perturbation introduced in Eq. (S31). The self-energy is evaluated at the photoelectron kinetic energy of ω_x-I_i (i.e., the photon energy minus the inner-shell binding energy).

S2.1 Approximation: Macroscopically Averaged Dielectric Function of the Solvent

Generally, for a microscopically inhomogeneous system, neither V_{sc} nor $\mathcal{M}(\mathcal{E})$ is diagonal in $\vec{k}$. In fact, EXAFS is sensitive to microscopic structure exactly for that reason. The key approximation in this work is that we continue to take into consideration the granularity of matter through V_{sc}, but for the self-energy of the photoelectron, we assume electrostatic interaction with a continuous dielectric medium. Neglecting the self-energy contribution from the parent atom, we employ the macroscopically averaged dielectric function of the host medium (in particular, water in this work), which is known experimentally, and we use it within the theoretical framework of the homogeneous electron gas for evaluating the required self-energy.

We note that by expanding the photoelectron propagator $G_{\vec{k}\vec{k}'}(\mathcal{E})$ to first order in V_{sc}, we recover the simplest model for describing EXAFS. If we treated $\mathcal{M}(\mathcal{E})$ also as a perturbation, which would be consistent with how $\mathcal{M}(\mathcal{E})$ is derived in the framework of many-body

perturbation theory, we would get an expansion of the form

$$\begin{aligned}\boldsymbol{G} &= \boldsymbol{G}^{(0)} + \boldsymbol{G}^{(0)}\boldsymbol{V}_{\rm sc}\boldsymbol{G}^{(0)} + \boldsymbol{G}^{(0)}\boldsymbol{\mathcal{M}}\boldsymbol{G}^{(0)} + \boldsymbol{G}^{(0)}\boldsymbol{V}_{\rm sc}\boldsymbol{G}^{(0)}\boldsymbol{V}_{\rm sc}\boldsymbol{G}^{(0)} + \boldsymbol{G}^{(0)}\boldsymbol{V}_{\rm sc}\boldsymbol{G}^{(0)}\boldsymbol{\mathcal{M}}\boldsymbol{G}^{(0)} \\ &\quad + \boldsymbol{G}^{(0)}\boldsymbol{\mathcal{M}}\boldsymbol{G}^{(0)}\boldsymbol{V}_{\rm sc}\boldsymbol{G}^{(0)} + \boldsymbol{G}^{(0)}\boldsymbol{\mathcal{M}}\boldsymbol{G}^{(0)}\boldsymbol{\mathcal{M}}\boldsymbol{G}^{(0)} + \ldots\,, \end{aligned} \tag{S33}$$

with

$$G^{(0)}_{\vec{k}\vec{k}'}(\mathcal{E}) = \left\langle \vec{k} \right| \frac{1}{\mathcal{E} - \left[-\frac{1}{2}\vec{\nabla}^2 - i\frac{\Gamma_i}{2}\right]} \left| \vec{k}' \right\rangle\,. \tag{S34}$$

[All matrices appearing in Eq. (S33) refer to the momentum representation. For example, $(\boldsymbol{G})_{\vec{k}\vec{k}'} = G_{\vec{k}\vec{k}'}$.] This means that if we truncated after the first order, i.e.,

$$\boldsymbol{G} \simeq \boldsymbol{G}^{(0)} + \boldsymbol{G}^{(0)}\boldsymbol{V}_{\rm sc}\boldsymbol{G}^{(0)} + \boldsymbol{G}^{(0)}\boldsymbol{\mathcal{M}}\boldsymbol{G}^{(0)}\,, \tag{S35}$$

the photoabsorption cross section would consist of two terms that show no EXAFS oscillations—those from $\boldsymbol{G}^{(0)}$ and $\boldsymbol{G}^{(0)}\boldsymbol{\mathcal{M}}\boldsymbol{G}^{(0)}$—and the "standard" EXAFS term from $\boldsymbol{G}^{(0)}\boldsymbol{V}_{\rm sc}\boldsymbol{G}^{(0)}$ (not entirely standard, because as defined earlier, $V_{\rm sc}$ includes the static interaction of the photoelectron with its parent atom). As a consequence, when using $V_{\rm sc} + \mathcal{M}$ as the perturbation, then, to first order, the EXAFS structure is not affected by $\mathcal{M}$. Hence, one would have to go at least to second order in such a framework.

S2.2 Perturbation Theory in the Scattering Potential

Even though there is no evident reason why $\mathcal{M}$ should be considered to be stronger than $V_{\rm sc}$, we aim to maintain the picture of individual scattering events of the photoelectron at the scattering atoms in the neighborhood of the absorbing atom. We will consider the dielectric medium a molasses-like substance and will thus employ in the following the strategy of including the self-energy in the unperturbed Green's function of the photoelectron. This makes at least formal sense, because the particular self-energy approximation that we use is diagonal in $\vec{k}$. Thus, within this picture, only $V_{\rm sc}$ is treated as a perturbation. In other

words, instead of the $G^{(0)}$ given in Eq. (S34), we employ the following zeroth-order Green's function

$$
\begin{aligned}
G^{(0)}_{\vec{k}\vec{k}'}(\mathcal{E}) &= \left\langle \vec{k} \middle| \frac{1}{\mathcal{E} - \left[-\frac{1}{2}\vec{\nabla}^2 + \mathcal{M}(\mathcal{E}) - i\frac{\Gamma_i}{2} \right]} \middle| \vec{k}' \right\rangle \\
&= \frac{1}{\mathcal{E} - \left[\frac{k^2}{2} + \mathcal{M}(k, \mathcal{E}) - i\frac{\Gamma_i}{2} \right]} \delta(\vec{k} - \vec{k}') , \qquad \text{(S36)}
\end{aligned}
$$

and use this to expand the photoelectron propagator of Eq. (S32) to first order in the perturbation V_{sc}.

Hence, we have

$$
\begin{aligned}
G_{\vec{k}\vec{k}'}(\mathcal{E} = \omega_x - I_i) &= G^{(0)}_{\vec{k}\vec{k}'}(\mathcal{E}) + \int d^3k_1 \int d^3k_2 \, G^{(0)}_{\vec{k}\vec{k}_1}(\mathcal{E}) \left\langle \vec{k}_1 \middle| V_{\mathrm{sc}} \middle| \vec{k}_2 \right\rangle G^{(0)}_{\vec{k}_2\vec{k}'}(\mathcal{E}) \\
&= \frac{1}{\mathcal{E} - \left[\frac{k^2}{2} + \mathcal{M}(k, \mathcal{E}) - i\frac{\Gamma_i}{2} \right]} \delta(\vec{k} - \vec{k}') \qquad \text{(S37)} \\
&\quad + \frac{1}{\mathcal{E} - \left[\frac{k^2}{2} + \mathcal{M}(k, \mathcal{E}) - i\frac{\Gamma_i}{2} \right]} \left\langle \vec{k} \middle| V_{\mathrm{sc}} \middle| \vec{k}' \right\rangle \frac{1}{\mathcal{E} - \left[\frac{k'^2}{2} + \mathcal{M}(k', \mathcal{E}) - i\frac{\Gamma_i}{2} \right]} ,
\end{aligned}
$$

with $\mathcal{E} = \omega_x - I_i$.

We insert this approximation to the photoelectron Green's function into the expression for the photoabsorption cross section, Eq. (S20), and obtain

$$
\begin{aligned}
\sigma_{\mathrm{abs}} &= -\frac{8\pi\alpha}{\omega_x} \int d^3k \int d^3k' \qquad \text{(S38)} \\
&\quad \times \mathrm{Im} \left\{ \left[G^{(0)}(k, \mathcal{E}) \delta(\vec{k} - \vec{k}') + G^{(0)}(k, \mathcal{E}) \left\langle \vec{k} \middle| V_{\mathrm{sc}} \middle| \vec{k}' \right\rangle G^{(0)}(k', \mathcal{E}) \right] \right. \\
&\quad \left. \times (\vec{k} \cdot \vec{\epsilon}^{\,*}) \left\langle \vec{k} \middle| i \right\rangle^* (\vec{k}' \cdot \vec{\epsilon}) \left\langle \vec{k}' \middle| i \right\rangle \right\} .
\end{aligned}
$$

Here, we employ the notation

$$
G^{(0)}(k, \mathcal{E}) = \frac{1}{\mathcal{E} - \left[\frac{k^2}{2} + \mathcal{M}(k, \mathcal{E}) - i\frac{\Gamma_i}{2} \right]} . \qquad \text{(S39)}
$$

Equations (S38) and (S39) correspond to Eqs. (8) and (9) in the main text.

S2.3 Self-energy of the Photoelectron in the Dielectric Solvent

In this subsection, we derive the self-energy of the photoelectron in the dissipative solvent by adopting the GW approximation together with using the optical potential, which follows the Hedin-Lundqvist approach.[S4,S5] Lundqvist calculated a complex optical potential for the photoelectron in terms of a self-energy that depends on the dielectric function $\varepsilon(q, \omega)$ of the environment. This continuous dielectric function effectively describes the interaction of the photoelectron with all other electrons in the host within a continuum approximation.

In order to determine the dielectric function, we use a parametrization of the solvent dielectric function either from measured experimental data and accurate fitting to, e.g., an extended Drude-Lorentz model,[S6,S7] or we may use dielectric functions calculated from quantum mechanics / molecular mechanics (QM/MM) simulations. Once the dielectric function is known, the self-energy can be calculated in terms of the fitting parameters of the parametrization, or, as a numerically determined result. With this, the EXAFS absorption coefficient can then be determined.

We shall use the GW approximation, which is the simplest that goes beyond the Hartree-Fock approximation. Then, the self-energy $\mathcal{M}$ is in general a functional of G, i.e., $\mathcal{M} = \mathcal{M}[G]$. The simplest approach is to expand $\mathcal{M}$ in a power series of the bare Coulomb interaction potential.[S4] However, such an expansion may diverge, which is the case for electrons in metals. Even in cases when it is convergent, its convergence rate rapidly becomes poor with increasing polarizability of the system. One common way to handle this problem is to make partial resummations to infinite order. The difficulty here is deciding on which partial summations to choose in order to obtain a systematic theory. Hedin[S4] developed a systematic expansion in terms of a screened potential $V(\vec{q}, \omega)$ rather than the bare Coulomb potential v_q using the Schwinger technique of functional derivatives.

In general, Hedin's self-energy reads

$$\mathcal{M}(\vec{k},\mathcal{E}) = i\int \frac{d^3q}{(2\pi)^3}\int_0^\infty \frac{d\omega}{2\pi} e^{-i\delta\omega} G(\vec{k}-\vec{q},\mathcal{E}-\omega)\Gamma(\vec{k},\mathcal{E};\vec{q},\omega)V(\vec{q},\omega), \tag{S40}$$

with G being the full Green's function. The function Γ involves the coupling to the infinite hierarchy of many-particle interactions. δ is a positive infinitesimal. The screened Coulomb potential $V(\vec{q},\omega)$ is defined as

$$V(\vec{q},\omega) = \frac{v_q}{\varepsilon(\omega,\vec{q})}\,, \tag{S41}$$

with

$$v_q = \frac{4\pi}{\vec{q}^2} \tag{S42}$$

being the bare Coulomb potential, and $\varepsilon(\omega,\vec{q})$ is the dielectric function. As before, $\vec{k}$ represents the three-dimensional momentum of the photoelectron, with $k = |\vec{k}|$ being its absolute value, and $\mathcal{E}$ is its energy. Moreover, $\vec{q}$ is the three-dimensional momentum vector of the electronic polarization wave existing in the solvent, while ω is its frequency.

S2.3.1 GW Approximation and Drude-Lorentz Dielectric Function

To proceed, we include all electronic Coulomb interaction effects in the dielectric function in a parametrized form. Moreover, we use the GW approximation and set $\Gamma(\vec{k},\mathcal{E};\vec{q},\omega)$ to 1, i.e., we stop the hierarchy after the first term. In addition, the full Green's function G is approximated by the Green's function for the noninteracting homogeneous electron gas in its ground state,

$$G_0(\vec{k},\mathcal{E}) = \frac{1}{\mathcal{E}-\frac{\vec{k}^2}{2}\pm i\delta}\,. \tag{S43}$$

Whether one uses $+i\delta$ or $-i\delta$ depends on the electron momentum considered: The ground state of the homogeneous electron gas is characterized by the Fermi momentum

$$k_F = (3\pi^2\rho)^{1/3}\,, \tag{S44}$$

where ρ is the electron number density. If $|\vec{k}| > k_F$, i.e., if the electron momentum is outside the Fermi sphere, then $+i\delta$ is required. Otherwise, if the electron momentum is inside the Fermi sphere, $-i\delta$ must be used. The zeroth-order Green's function G_0 is introduced here exclusively in order to determine the self-energy. It should not be confused with the $G^{(0)}$ defined in Eq. (S39).

Overall, we use the GW-approximated self-energy

$$\mathcal{M}(\vec{k}, \mathcal{E}) = 4\pi i \int \frac{d^3q}{(2\pi)^3} \int_0^\infty \frac{d\omega}{2\pi} e^{-i\delta\omega} G_0(\vec{k}-\vec{q}, \mathcal{E}-\omega) \frac{1}{\vec{q}^2} \frac{1}{\varepsilon(\omega, \vec{q})} . \tag{S45}$$

The dielectric function can be taken into consideration either as a numerical-data array or as a chosen analytic function. In the present work, we shall use a parametrized Drude-Lorentz-type expression

$$\varepsilon(\omega, \vec{q}) = \frac{\omega_0^2(\vec{q}) - \omega^2 - i\omega\gamma(\vec{q}) + \omega_p^2(\vec{q})}{\omega_0^2(\vec{q}) - \omega^2 - i\omega\gamma(\vec{q})} . \tag{S46}$$

A common way to include the $\vec{q}$ dependence is by utilizing $\vec{q}$-dependent parameters $\gamma(\vec{q}), \omega_p(\vec{q})$, and $\omega_0(\vec{q})$. See also Refs. S4,S5. Note that Lundqvist uses the plasmon pole approximation,[S5] which assumes that there is a sharp pole of the self-energy at the plasma frequency (see Sec. S3.3 for further details). This gives rise to a formalism in which the dielectric function is assumed real. In the approach pursued here, we obtain a rather broad (in frequency space) and complex-valued dielectric function of the water solvent (in addition to a more complicated $\vec{q}$ dependence), which prevents us from using the plasmon pole approximation.

Collecting all parts, we obtain $\mathcal{M}(\vec{k}, \mathcal{E})$ as

$$\begin{aligned}
\mathcal{M}(\vec{k}, \mathcal{E}) &= 4\pi i \int \frac{d^3q}{(2\pi)^3} \int_0^\infty \frac{d\omega}{2\pi} \left(\frac{\Theta(|\vec{k}-\vec{q}| - k_F)}{\mathcal{E} - \omega - \frac{(\vec{k}-\vec{q})^2}{2} + i\delta} + \frac{\Theta(k_F - |\vec{k}-\vec{q}|)}{\mathcal{E} - \omega - \frac{(\vec{k}-\vec{q})^2}{2} - i\delta} \right) \frac{1}{\vec{q}^2} \\
&\quad \times \frac{\omega_0^2(\vec{q}) - \omega^2 - i\omega\gamma(\vec{q})}{\omega_0^2(\vec{q}) - \omega^2 - i\omega\gamma(\vec{q}) + \omega_p^2(\vec{q})} \\
&= \mathcal{M}_1(\vec{k}, \mathcal{E}) + \mathcal{M}_2(\vec{k}, \mathcal{E}) ,
\end{aligned} \tag{S47}$$

where

$$\mathcal{M}_1(\vec{k},\mathcal{E}) = 4\pi i \int \frac{d^3q}{(2\pi)^3}\frac{1}{\vec{q}^2}\int_0^\infty \frac{d\omega}{2\pi}\frac{\Theta(|\vec{k}-\vec{q}|-k_F)}{\omega-\left[\mathcal{E}-\frac{(\vec{k}-\vec{q})^2}{2}+i\delta\right]}\frac{\omega_0^2(\vec{q})-\omega^2-i\omega\gamma(\vec{q})}{\omega^2+i\omega\gamma(\vec{q})-(\omega_0^2(\vec{q})+\omega_p^2(\vec{q}))}\,, \tag{S48}$$

$$\mathcal{M}_2(\vec{k},\mathcal{E}) = 4\pi i \int \frac{d^3q}{(2\pi)^3}\frac{1}{\vec{q}^2}\int_0^\infty \frac{d\omega}{2\pi}\frac{\Theta(k_F-|\vec{k}-\vec{q}|)}{\omega-\left[\mathcal{E}-\frac{(\vec{k}-\vec{q})^2}{2}-i\delta\right]}\frac{\omega_0^2(\vec{q})-\omega^2-i\omega\gamma(\vec{q})}{\omega^2+i\omega\gamma(\vec{q})-(\omega_0^2(\vec{q})+\omega_p^2(\vec{q}))}\,. \tag{S49}$$

From now on, we assume spatial isotropy of the dielectric medium, which implies that the dielectric function depends only on the magnitude $q = |\vec{q}|$ of the transferred momentum and not on its direction. Hence, $\omega_0(\vec{q}) = \omega_0(q)$, $\omega_p(\vec{q}) = \omega_p(q)$, and $\gamma(\vec{q}) = \gamma(q)$. With this assumption, Eq. (11) in the main text follows from Eq. (S46).

S3 Parametrization of the Dielectric Function of Water

In general, the dielectric function is complex and is written as

$$\varepsilon(\omega, q) = \varepsilon_1(\omega, q) + i\varepsilon_2(\omega, q)\,. \tag{S50}$$

It is a function of the energy ω and the momentum q transferred in the excitation of the dielectric solvent. Often, it is expressed in terms of the energy loss function

$$\mathrm{Im}\left(-\frac{1}{\varepsilon(\omega, q)}\right) = \frac{\varepsilon_2(\omega, q)}{|\varepsilon(\omega, q)|^2}\,. \tag{S51}$$

For the case of water as a dielectric medium, it has been accurately measured experimentally by neutron scattering experiments, and also parametrized functional forms exist. Below, we shall use an advanced form of Refs. S6,S7, but also fit this form to a simplified Drude-Lorentz

form, the latter allowing us to carry out frequency integrals explicitly when evaluating the electronic self-energy.

S3.1 Parametrization of Emfietzoglou et al.

From a comparison with experimental data, Emfietzoglou *et al.*[S6,S7] obtained an accurate parametrization of the dielectric loss function of liquid water in terms of a linear superposition of Drude-Lorentz (for ionizations) and derivative-Drude-Lorentz (for excitations) functions. Their expressions for the real and imaginary parts of the dielectric function are given by

$$\begin{aligned}\varepsilon_1(E,q) &= 1+E_p^2\sum_j^{\text{ioniz}}\frac{f_j^{\text{ioniz}}(q)[E_j^{\text{ioniz}}(q)^2-E^2]}{[E_j^{\text{ioniz}}(q)^2-E^2]^2+[\gamma_j^{\text{ioniz}}(q)E]^2}\\ &\quad+E_p^2\sum_j^{\text{excit}}\frac{f_j^{\text{excit}}(q)[\gamma_j^{\text{excit}}(q)^2-E^2][(E_j^{\text{excit}\,2}-E^2)^2+3(\gamma_j^{\text{excit}}(q)E)^2]}{\left[(E_j^{\text{excit}\,2}-E^2)^2+(\gamma_j^{\text{excit}}(q)E)^2\right]^2}, \quad \text{(S52)}\\ \varepsilon_2(E,q) &= E_p^2\sum_j^{\text{ioniz}}\frac{f_j^{\text{ioniz}}(q)\gamma_j^{\text{ioniz}}(q)E}{[E_j^{\text{ioniz}}(q)^2-E^2]^2+[\gamma_j^{\text{ioniz}}(q)E]^2}\\ &\quad+E_p^2\sum_j^{\text{excit}}\frac{2f_j^{\text{excit}}(q)\gamma_j^{\text{excit}}(q)^3E^3}{\left[(E_j^{\text{excit}\,2}-E^2)^2+(\gamma_j^{\text{excit}}(q)E)^2\right]^2}, \quad \text{(S53)}\end{aligned}$$

where E_p is the nominal free-electron plasma energy of liquid water (~ 21.46 eV). $f_j^{\text{ioniz}}(q)$ ($f_j^{\text{excit}}(q)$), $\gamma_j^{\text{ioniz}}(q)$ ($\gamma_j^{\text{excit}}(q)$) and $E_j^{\text{ioniz}}(q)$ (E_j^{excit}) are the oscillator strength, damping energy, and transition-energy coefficients. With different dispersion relations used for the excitation levels and the ionization shells, the momentum dependence is directly introduced into these Drude-Lorentz model coefficients. Moreover, we have

$$\begin{aligned}&f_j^{\text{ioniz}}(q)=f_j^{\text{ioniz}}\frac{Z-\sum_j^{\text{excit}}f_j^{\text{excit}}(q)}{Z-\sum_j^{\text{excit}}f_j^{\text{excit}}},\ f_j^{\text{excit}}(q)=f_j^{\text{excit}}\left[e^{-a_jq^2}+b_jq^2e^{-c_jq^2}\right],\\ &\gamma_j^{\text{ioniz}}(q)=\gamma_j^{\text{ioniz}}+\beta_1(\text{Ry}q)+\beta_2(\text{Ry}q^2),\ \gamma_j^{\text{excit}}(q)=\gamma_j^{\text{excit}}+aq+bq^2, \quad \text{(S54)}\\ &E_j^{\text{ioniz}}(q)=E_j^{\text{ioniz}}+a_{\text{emp}}(q)\text{Ry}q^2,\ a_{\text{emp}}(q)=1-e^{-cq^d},\end{aligned}$$

where $Z = 10$ for water, with the empirical coefficients $a = 10$ and $b = 6$ (with q in atomic units) and with $\beta_1 = 0.735$, $\beta_2 = 0.441$, $c = 1.5$, and $d = 0.4$ (q in atomic units). a_j, b_j, and c_j are also empirical parameters. f_j^{ioniz} (f_j^{excit}), γ_j^{ioniz} (γ_j^{excit}), and E_j^{ioniz} (E_j^{excit}) are the values of the Drude-Lorentz-model coefficients when $q = 0$, which are determined from fitting dielectric response data at the optical limit for liquid water,[S6] see Fig. S1 for a schematic overview. Moreover, we refer to Refs. S8,S9 for further details. For the benefit of the reader,

	oscillator strength		damping energy		Transition-energy coefficients	
	$q = 0$	$q \neq 0$	$q = 0$	$q \neq 0$	$q = 0$	$q \neq 0$
Ionization shell	f_j^{ioniz}	f_j^{ioniz}(q)	γ_j^{ioniz}	γ_j^{ioniz}(q)	E_j^{ioniz}	E_j^{ioniz}(q)
Excitation shell	f_j^{excit}	f_j^{excit}(q)	γ_j^{excit}	γ_j^{excit}(q)	E_j^{excit}	

Figure S1: Overview of the Drude-Lorentz-model coefficients used in Ref. S6.

we reproduce the remaining parameters in Tab. S1 from Ref. S6.

Table S1: The Drude-Lorentz model parameters of Emfietzoglou et al.[S6] Reproduced from Ref. S6 with permission. Copyright 2025 Radiation Research Society.

Transition, j	E_j (eV)	γ_j (eV)	f_j
Excitations			
1	8.10	1.90	0.0045
2	10.10	1.95	0.0046
3	12.00	2.94	0.0030
4	13.51	5.06	0.0190
5	14.41	2.64	0.0050
Ionizations			
6	16.30	14.00	0.2300
7	17.25	10.91	0.1600
8	28.00	27.38	0.1890
9	42.00	28.68	0.2095
10	450	360	0.3143

We have reproduced the dielectric response function data of this extended theoretical Drude-Lorentz-type model. Moreover, for $q = 0$, we have compared the energy loss function to the more recent inelastic-x-ray-scattering data of Hayashi *et al.*[S10] The comparison is shown in Fig. S2. The agreement is not perfect, but the parametrization of Emfietzoglou *et al.* does capture the key features of the experimental data.

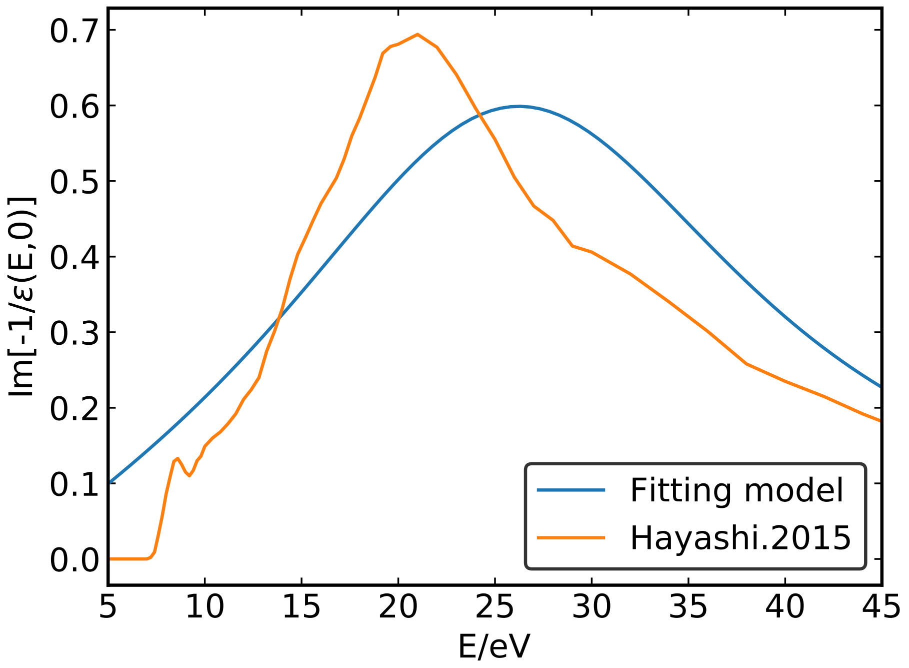

Figure S2: Energy-loss function of liquid water for $q = 0$: (blue line) parametrization of Emfietzoglou *et al.*;[S6,S8,S9] (orange line) measured optical energy-loss function of Hayashi *et al.*[S10] Data reproduced using parameters from Ref. S6 with the permission of the Radiation Research Society. Copyright 2025 Radiation Research Society. Data reproduced from Ref. S10. Copyright 2015 American Chemical Society.

S3.2 Effective Drude-Lorentz Parametrization

The model of Emfietzoglou *et al.* is still too complicated for the analytic evaluation of the frequency integral in the electronic self-energy in Eq. (S45). For further simplification, we propose a fitting model for the dielectric loss function in terms of a Drude-Lorentz form

according to

$$\mathrm{Im}\left(-\frac{1}{\varepsilon(\omega,q)}\right) = \frac{B_0(q)\omega}{[\omega^2 - B_1(q)^2]^2 + [\omega B_2(q)]^2}$$
$$= \frac{\omega_p(q)^2\gamma(q)\omega}{[\omega_0(q)^2 + \omega_p(q)^2 - \omega^2]^2 + [\omega\gamma(q)]^2}\,, \tag{S55}$$

where $B_0(q)$, $B_1(q)$, and $B_2(q)$ are q-dependent fitting coefficients. By fitting with the model of Emfietzoglou *et al.* described in the previous subsection, we obtain for the coefficients (in atomic units)

$$\begin{aligned} B_0(q) &= 0.24995q^2 - 0.34207q + 0.79275\,, \\ B_1(q) &= 0.54463q^2 - 0.32634q + 1.10691\,, \\ B_2(q) &= 0.31715q^2 + 0.04447q + 1.13079\,. \end{aligned} \tag{S56}$$

Expressed in terms of the functions introduced in Eq. (S46), these fitting coefficients are given by

$$\begin{aligned} B_0(q) &= \omega_p(q)^2\gamma(q)\,, \\ B_1(q) &= \sqrt{\omega_0(q)^2 + \omega_p(q)^2}\,, \\ B_2(q) &= \gamma(q)\,. \end{aligned} \tag{S57}$$

Conversely, we have

$$\begin{aligned} \omega_p(q) &= \sqrt{B_0(q)/B_2(q)}\,, \\ \omega_0(q) &= \sqrt{B_1^2(q) - B_0(q)/B_2(q)}\,, \\ \gamma(q) &= B_2(q)\,. \end{aligned} \tag{S58}$$

The comparison of the energy-loss function between the effective Drude-Lorentz model

and the parametrized full model of Emfietzoglou *et al.* (Fig. S3) shows that our simplified fitting model works rather well.

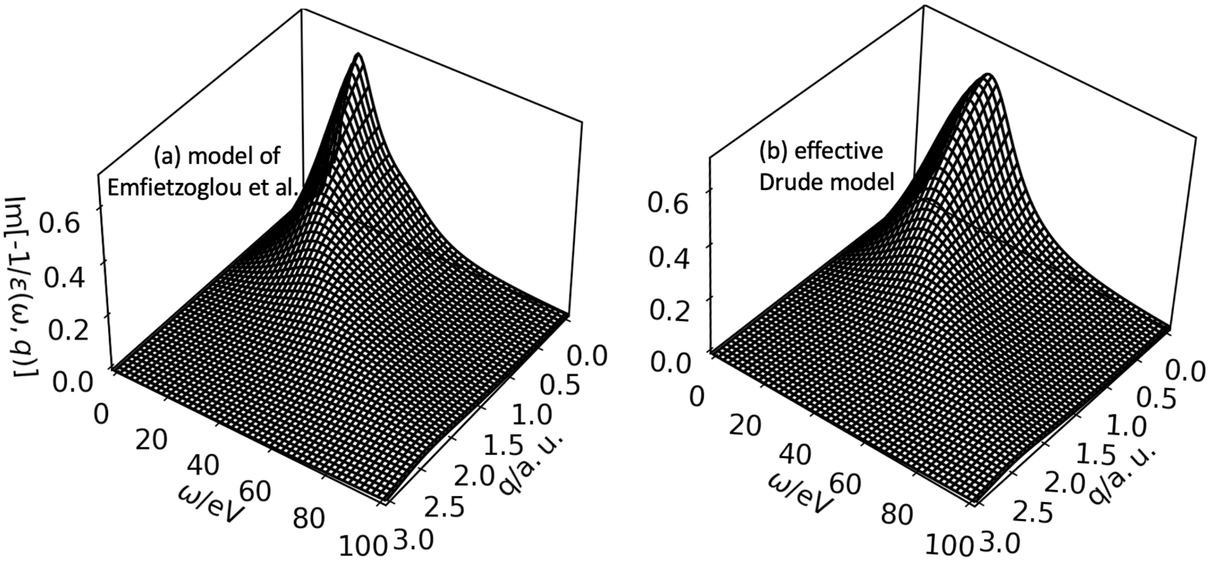

Figure S3: Energy-loss function of liquid water at room temperature: (a) reproduced model of Emfietzoglou *et al.*,[S6,S8,S9] (b) effective Drude-Lorentz model (a.u., atomic units). Data reproduced using parameters from Ref. S6 with the permission of the Radiation Research Society. Copyright 2025 Radiation Research Society.

S3.3 Comparison to the Plasmon-Pole Approximation

A common approximation to the complex dielectric function is the (single) plasmon pole model.[S4,S5,S11] It assumes that the dominant response of a system of electrons in a metal to an electric field occurs by a collective excitation mode, the plasmon mode. It responds at a single constant frequency ω_p, the plasma frequency, and yields to the dielectric function

$$\varepsilon_{\mathrm{p.p.}}(\omega, q) = \frac{\Omega^2(q) - \omega^2}{\Omega^2(q) - \omega_p^2 - \omega^2 - 2i\omega\delta}\,, \tag{S59}$$

with an infinitesimal $\delta > 0$, with $\Omega^2(q) = \omega_p^2[1 + q^2/\kappa^2]$. Here, κ is the Thomas-Fermi screening wave number.[S11] The dielectric loss function follows as

$$\mathrm{Im}\left(-\frac{1}{\varepsilon(\omega, q)}\right) = \frac{\pi}{2}\frac{\omega_p^2}{\Omega(q)}\left[\delta(\omega - \Omega(q)) - \delta(\omega + \Omega(q))\right] . \tag{S60}$$

This approximate form of the dielectric function represents the collective plasma mode with the real frequency $\Omega(q)$ and thereby does not include any form of damping. The single-plasmon-pole approximation is appropriate for electrons in metals with a continuous energy band and with sharp plasmon peaks in the inverse dielectric function, e.g., Al, i.e., whenever nearly free electron gas models apply. Put differently, in solvents with strong damping, localized polarization excitations, and no relevant collective modes, the single-pole approximation is not realistic. Even at low energies for transition metals, insulators, and molecules with more complex loss spectra, it often loses accuracy, and in practice, often gives an unphysical structure to the self-energy near ω_p.[S13] In contrast, damping of the photoelectron in the solvent is significant, which can be seen quantitatively from Eqs. (S56) and (S58), where the damping parameter $\gamma(0) \simeq \omega_p(0) \simeq \omega_0(0)$. Put differently, the damping of the Drude-Lorentz oscillator is comparable to its oscillation frequency.

S4 Evaluation of the Dissipative Photoelectron Self-energy

Using the Drude-Lorentz-parametrized form of the dielectric function, we can evaluate the photoelectron self-energy terms, Eqs. (S48) and (S49).

S4.1 Frequency Integrals

First, we carry out the ω integration in $\mathcal{M}_1(\vec{k},\mathcal{E})$. The integrand has three poles, which are given by

$$\begin{aligned}
\omega_1(\vec{q}) &= \mathcal{E} - \frac{(\vec{k}-\vec{q})^2}{2} + i\delta\,, \\
\omega_{2/3}(\vec{q}) &= \frac{-i\gamma(\vec{q}) \pm \sqrt{-\gamma^2(\vec{q}) + 4(\omega_0^2(\vec{q}) + \omega_p^2(\vec{q}))}}{2}\,.
\end{aligned} \tag{S61}$$

We consider a contour integration in the lower complex plane. ω_1 does not contribute to the integral, since it lies in the upper complex plane. The remaining residues are

$$\begin{aligned}
\mathrm{Res}_2 &= \frac{\Theta(|\vec{k}-\vec{q}|-k_F)}{\omega_2(\vec{q})-\omega_1}\frac{\omega_0^2(\vec{q})-\omega_2^2(\vec{q})-i\omega_2(\vec{q})\gamma(\vec{q})}{\omega_2(\vec{q})-\omega_3(\vec{q})}\,, \\
\mathrm{Res}_3 &= \frac{\Theta(|\vec{k}-\vec{q}|-k_F)}{\omega_3(\vec{q})-\omega_1}\frac{\omega_0^2(\vec{q})-\omega_3^2(\vec{q})-i\omega_3(\vec{q})\gamma(\vec{q})}{\omega_3(\vec{q})-\omega_2(\vec{q})}\,.
\end{aligned} \tag{S62}$$

This gives

$$\mathcal{M}_1(\vec{k},\mathcal{E}) = 4\pi \int \frac{d^3q}{(2\pi)^3}\frac{1}{\vec{q}^2}(\mathrm{Res}_2 + \mathrm{Res}_3) = \mathcal{M}_{1,1}(\vec{k},\mathcal{E}) + \mathcal{M}_{1,2}(\vec{k},\mathcal{E})\,. \tag{S63}$$

Next, we calculate the ω integral in $\mathcal{M}_2(\vec{k},\mathcal{E})$. The integrand also has three poles. These are given by

$$\begin{aligned}
\omega_1'(\vec{q}) &= \mathcal{E} - \frac{(\vec{k}-\vec{q})^2}{2} - i\delta\,, \\
\omega_{2/3}'(\vec{q}) &= \frac{-i\gamma(\vec{q}) \pm \sqrt{-\gamma^2(\vec{q}) + 4(\omega_0^2(\vec{q}) + \omega_p^2(\vec{q}))}}{2}\,.
\end{aligned} \tag{S64}$$

Again, we perform a contour integration in the lower complex plane. In this case, all three

poles contribute to this integral and we find the residues

$$\begin{aligned}
\mathrm{Res}_1' &= \Theta(k_F - |\vec{k}-\vec{q}|)\frac{\omega_0^2(\vec{q}) - \omega_2'^2(\vec{q}) - i\omega_2'(\vec{q})\gamma(\vec{q})}{(\omega_1' - \omega_2'(\vec{q}))(\omega_1' - \omega_3'(\vec{q}))}\,, \\
\mathrm{Res}_2' &= \frac{\Theta(k_F - |\vec{k}-\vec{q}|)}{\omega_2'(\vec{q}) - \omega_1'}\frac{\omega_0^2(\vec{q}) - \omega_2'^2(\vec{q}) - i\omega_2'(\vec{q})\gamma(\vec{q})}{\omega_2'(\vec{q}) - \omega_3'(\vec{q})}\,, \\
\mathrm{Res}_3' &= \frac{\Theta(k_F - |\vec{k}-\vec{q}|)}{\omega_3'(\vec{q}) - \omega_1'}\frac{\omega_0^2(\vec{q}) - \omega_3'^2(\vec{q}) - i\omega_3'(\vec{q})\gamma(\vec{q})}{\omega_3'(\vec{q}) - \omega_2'(\vec{q})}\,,
\end{aligned} \tag{S65}$$

yielding

$$\begin{aligned}
\mathcal{M}_2(\vec{k},\mathcal{E}) &= 4\pi i \int \frac{d^3q}{(2\pi)^3}\frac{1}{\vec{q}^2}\frac{1}{2\pi}(-2\pi i)(\mathrm{Res}_1' + \mathrm{Res}_2' + \mathrm{Res}_3') \\
&= -4\pi \int \frac{d^3q}{(2\pi)^3}\frac{\Theta(k_F - |\vec{k}-\vec{q}|)}{\vec{q}^2}\,.
\end{aligned} \tag{S66}$$

Note that this term in the photoelectron self-energy does not depend on the parameters appearing in the dielectric function. In fact, it equals the well-known exchange potential in the homogeneous electron gas.[S3] In order to avoid the double-counting problem mentioned in Sec. S2, we drop the term $\mathcal{M}_2$ from the self-energy and do not consider it any further.

S4.2 Momentum Integral

Now we carry out the $\vec{q}$ integration in $\mathcal{M}_1(\vec{k},\mathcal{E})$. Because both $\omega_2(\vec{q})$ and $\omega_3(\vec{q})$ have a finite imaginary part, $i\delta$ can be dropped.

For a given $\vec{k}$, $\Theta(|\vec{q}-\vec{k}| - k_F)$ gives a nonzero contribution only outside a sphere in $\vec{q}$ space centered at $\vec{q} = \vec{k}$ with radius k_F. Therefore, let us switch to the integration variable

$$\vec{q}\,' = \vec{q} - \vec{k}\,. \tag{S67}$$

Now, we can recenter the sphere and find

$$\begin{aligned}\mathcal{M}_{1,1}(\vec{k},\mathcal{E}) &= \frac{1}{2\pi^2}\int d^3q'\frac{\omega_0^2(|\vec{q}\,'+\vec{k}|)-\omega_2^2(|\vec{q}\,'+\vec{k}|)-i\omega_2(|\vec{q}\,'+\vec{k}|)\gamma(|\vec{q}\,'+\vec{k}|)}{\omega_2(|\vec{q}\,'+\vec{k}|)-\omega_3(|\vec{q}\,'+\vec{k}|)}\\ &\quad\times\frac{1}{(\vec{q}\,'+\vec{k})^2}\frac{\Theta(q'-k_F)}{\omega_2(|\vec{q}\,'+\vec{k}|)-\left[\mathcal{E}-\frac{q'^2}{2}\right]}\\ &= \frac{1}{\pi}\int_{k_F}^{\infty}dq\int_{-1}^{1}du\frac{\omega_0^2(|\vec{q}+\vec{k}|)-\omega_2^2(|\vec{q}+\vec{k}|)-i\omega_2(|\vec{q}+\vec{k}|)\gamma(|\vec{q}+\vec{k}|)}{\omega_2(|\vec{q}+\vec{k}|)-\omega_3(|\vec{q}+\vec{k}|)}\\ &\quad\times\frac{1}{q^2+k^2+2qku}\frac{q^2}{\omega_2(|\vec{q}+\vec{k}|)-\left[\mathcal{E}-\frac{q^2}{2}\right]}\,, \end{aligned}\tag{S68}$$

where

$$u=\cos\theta \tag{S69}$$

and

$$|\vec{q}+\vec{k}|=\sqrt{q^2+k^2+2qku}\,. \tag{S70}$$

After the second equality sign in Eq. (S68), we dropped the prime of q'.

Similarly, we find

$$\begin{aligned}\mathcal{M}_{1,2}(\vec{k},\mathcal{E}) &= \frac{1}{\pi}\int_{k_F}^{\infty}dq\int_{-1}^{1}du\frac{\omega_0^2(|\vec{q}+\vec{k}|)-\omega_3^2(|\vec{q}+\vec{k}|)-i\omega_3(|\vec{q}+\vec{k}|)\gamma(|\vec{q}+\vec{k}|)}{\omega_3(|\vec{q}+\vec{k}|)-\omega_2(|\vec{q}+\vec{k}|)}\\ &\quad\times\frac{1}{q^2+k^2+2qku}\frac{q^2}{\omega_3(|\vec{q}+\vec{k}|)-\left[\mathcal{E}-\frac{q^2}{2}\right]}\,. \end{aligned}\tag{S71}$$

In this representation, $\mathcal{M}_1$ depends only on the magnitude of $\vec{k}$, i.e., $\mathcal{M}_1(\vec{k},\mathcal{E})=\mathcal{M}_1(k,\mathcal{E})$. Hence, $\mathcal{M}(\vec{k},\mathcal{E})=\mathcal{M}(k,\mathcal{E})$. Moreover, it is easy to show that $\mathcal{M}(-k,\mathcal{E})=\mathcal{M}(k,\mathcal{E})$.

S4.3 Fermi Momentum in Water

To evaluate the electronic self-energy, we need to fix the Fermi momentum k_F in water. We determine k_F from the macroscopic valence electron density in water, by using Eq. (S44). To choose the electrons that we include in the density of the homogeneous electron gas used

in our approach, we consider the electronic configuration of the 8 electrons of oxygen, i.e., $1s^2 2s^2 2p^4$. The $1s$ electrons of oxygen are tightly bound and do not fit into the picture of a homogeneous electron gas. Hence, we start to count from the $2s$ electrons and have then 6 electrons. Together with the two electrons of hydrogen, we have 8 valence electrons per H_2O molecule. Liquid water has a mass density of 1 kg/l. The mass of a water molecule is 18 atomic mass units (a.m.u.), i.e., $18 \times 1.66 \times 10^{-27}$ kg $= 2.99 \times 10^{-26}$ kg. This yields a mean volume per water molecule in liquid water of $V_{\mathrm{H_2O}} = 2.99 \times 10^{-26}\,\mathrm{l} = 29.88\,\text{Å}^3 = 202\, a_0^3$, a_0 being the Bohr radius. Hence, $\rho = 8/202 = 0.0396$ in atomic units. Thus, using Eq. (S44), we find

$$k_F = (3\pi^2 \rho)^{1/3} = 1.055\,\mathrm{a.u.} \tag{S72}$$

With this, we may determine the width of the valence band in water according to

$$E_{\mathrm{bw}} = \frac{k_F^2}{2} = 0.55713\,\mathrm{a.u.}, \tag{S73}$$

corresponding to 15.16 eV. Of course, this picture treats the valence electrons in water as if they formed a homogeneous electron gas in its ground state. Interestingly, this picture is consistent with the width of the valence band in liquid water, which extends from $2\mathrm{a}_1$ to $1\mathrm{b}_1$ over approximately 20 eV.[S12] Certainly, the valence electrons are not free, but exist in a spatially inhomogeneous attractive potential landscape. Yet, we find a decent agreement.

S4.4 Numerical Evaluation of the Photoelectron Self-energy in the GW Approximation

Within the framework adopted here, it would be inconsistent to allow arbitrarily high momentum transfer to the medium, and then to assume that the response of the medium is the same at all momentum transfers, which is clearly not the case. Therefore, introducing a momentum-transfer cutoff q_{max} is appropriate. As a matter of fact, the description in terms of the dielectric is a low-energy/long-wavelength (classical) picture of the solvent. Hence, the

theory in the present form is restricted implicitly to low-energy excitations of the dielectric. Thus, we replace the upper limit ∞ in the q integral in Eqs. (S68) and (S71) by $q_{\max}$. We set $q_{\max} = 0.997\sqrt{2\mathcal{E}}$, where $\mathcal{E}$ is given by kinetic energy of the photoelectron, $\mathcal{E} = \omega_x - I_i$. On this basis, we perform the remaining integrations in the self-energy $\mathcal{M}(k,\mathcal{E}) = \mathcal{M}_1(k,\mathcal{E})$ numerically. For simplification, we set $k = \sqrt{2\mathcal{E}}$, so that the self-energy becomes a function of $\mathcal{E}$ only.

The results for the real and imaginary parts of $\mathcal{M}(k = \sqrt{2\mathcal{E}}, \mathcal{E})$ for water are shown in Fig. S4. We find that the imaginary part of the self-energy is always less than zero. This is consistent with an irreversible loss of one-particle weight in the many-body wave function as a function of time.

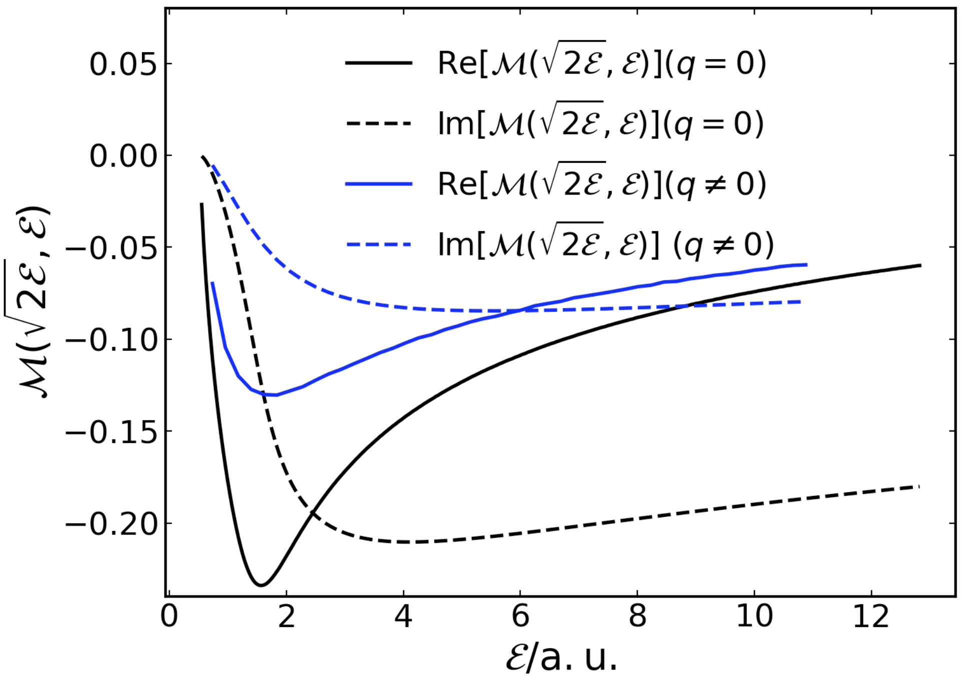

Figure S4: The dependence of the dissipative photoelectron self-energy $\mathcal{M}(k = \sqrt{2\mathcal{E}}, \mathcal{E})$ on $\mathcal{E}$ (in a.u.) for the case of the dielectric function of water. The blue lines show the result when a finite momentum transfer is taken into consideration. The black lines show the result when neglecting the q dependence in the dielectric function and simply using its value at $q = 0$. Solid lines: real part, dashed lines: imaginary part.

S4.5 Self-energy for $q = 0$

In this subsection, we briefly discuss the photoelectron self-energy when ignoring the q dependence of the dielectric function. Hence, we consider

$$\varepsilon(\omega) = \varepsilon(\omega, \vec{q} = 0) = \frac{\omega_0^2 - \omega^2 - i\omega\gamma + \omega_p^2}{\omega_0^2 - \omega^2 - i\omega\gamma}\,, \tag{S74}$$

where $\omega_0 = \omega_0(0)$, $\gamma = \gamma(0)$, and $\omega_p = \omega_p(0)$. The momentum integrations simplify slightly and one eventually finds

$$\begin{aligned} \mathcal{M}_1(k, \mathcal{E}) &= \frac{1}{k\pi} f_2 \int_{k_F}^{\infty} dq \left[\frac{1}{\omega_2 - \left[\mathcal{E} - \frac{q^2}{2}\right]} - \frac{1}{\omega_3 - \left[\mathcal{E} - \frac{q^2}{2}\right]} \right] q \ln\left(\left| \frac{q+k}{q-k} \right| \right) \\ &= \frac{1}{k\pi} f_2 \int_{k_F}^{\infty} dq \left[\frac{1}{\omega_2 - \left[\mathcal{E} - \frac{q^2}{2}\right]} + \frac{1}{\omega_2^* + \left[\mathcal{E} - \frac{q^2}{2}\right]} \right] q \ln\left(\left| \frac{q+k}{q-k} \right| \right)\,, \end{aligned} \tag{S75}$$

where

$$f_2 = \frac{\omega_0^2 - \omega_2^2 - i\omega_2\gamma}{\omega_2 - \omega_3}\,. \tag{S76}$$

The numerical result is shown in Fig. S4, which demonstrates the importance of capturing the dependence of the dielectric function on the momentum transfer to the dielectric medium.

S5 Validation of the Dissipative Photoelectron Self-energy

In order to assess the accuracy of the self-energy determined in Sec. S4, we compute the inelastic mean free path (IMFP) of an electron in water. When the photoelectron propagates through the solvent, it experiences inelastic energy losses due to polarization excitations in the dielectric medium. The intensity of the electron wave diminishes exponentially with $I(d) = I_0 e^{-d/\lambda}$, where d is the distance travelled and λ is the IMFP. Since the photoelectron self-energy $\mathcal{M}(k, \mathcal{E})$ describes those losses, the IMFP is determined by the self-energy

according to[S13]

$$\lambda = \sqrt{\frac{\mathcal{E}}{2}} \frac{1}{\left|\mathrm{Im}\left[\mathcal{M}(k=\sqrt{2\mathcal{E}}, \mathcal{E})\right]\right|}, \tag{S77}$$

where $\mathcal{E}$ is the kinetic energy of the photoelectron. Using the numerical evaluation of the dissipative photoelectron self-energy discussed in Sec. S4, we determine the IMFP of a photoelectron in water and compare the result with that obtained from accurate calculations.[S14] The comparison presented in Fig. S5 demonstrates that in the energy range of relevance to EXAFS (100 eV or higher), there is quite good agreement with the reference data.

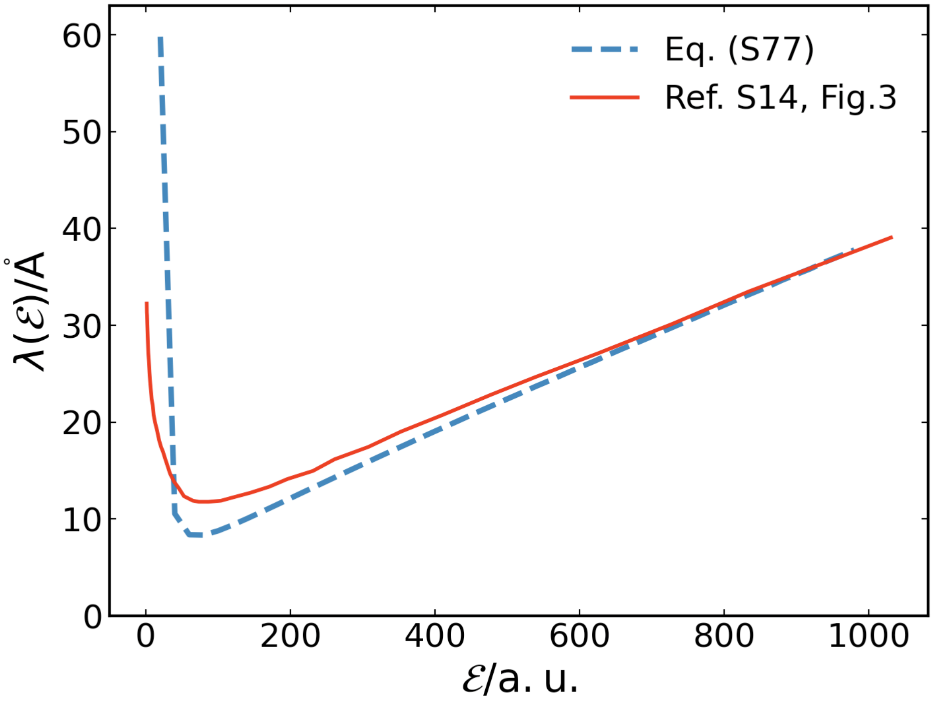

Figure S5: Calculated IMFP based on the dissipative photoelectron self-energy in GW approximation with the parametrized dielectric function of water (blue dashed line) in comparison with results from Ref. S14 (red solid line). Copyright IOP Publishing. Reproduced from Ref. S14 with permission. All rights reserved.

S6 Evaluation of the Absorption Cross Section

We now evaluate the absorption cross section σ_{abs} in Eq. (S38). In our model, this is simplified by the following observations: For K-shell photoabsorption, $\left\langle \vec{k} \middle| i \right\rangle^*$ and $\left\langle \vec{k}' \middle| i \right\rangle$ depend only

on $k = |\vec{k}|$ and $k' = |\vec{k}'|$, respectively. For the product $(\vec{k} \cdot \vec{\epsilon}^*)(\vec{k}' \cdot \vec{\epsilon})$, we use that (i) the x-rays normally employed for EXAFS are linearly polarized, and (ii) we focus on disordered systems with a solvent environment. Hence, we may replace $(\vec{k} \cdot \vec{\epsilon}^*)(\vec{k}' \cdot \vec{\epsilon})$ with

$$\begin{aligned} & \frac{1}{4\pi} \int d\Omega \, \{k_x \sin\vartheta \cos\varphi + k_y \sin\vartheta \sin\varphi + k_z \cos\vartheta\} \\ & \times \left\{k'_x \sin\vartheta \cos\varphi + k'_y \sin\vartheta \sin\varphi + k'_z \cos\vartheta\right\} \\ = \; & \frac{1}{3} (\vec{k} \cdot \vec{k}') \,. \end{aligned} \tag{S78}$$

The zeroth-order Green's functions in Eq. (S38) depend, as a consequence of the choice we have made, only on k and k', not on the vectors $\vec{k}$ and $\vec{k}'$.

Because of the spherical symmetry assumed for the atomic scattering potential, we can write $\left\langle \vec{k} \middle| V_n \middle| \vec{k}' \right\rangle$ as

$$\begin{aligned} \left\langle \vec{k} \middle| V_n \middle| \vec{k}' \right\rangle &= \frac{1}{(2\pi)^3} \int d^3x \, e^{i(\vec{k}' - \vec{k}) \cdot \vec{x}} V_n(|\vec{x} - \vec{R}_n|) \\ &= e^{i(\vec{k}' - \vec{k}) \cdot \vec{R}_n} \frac{1}{(2\pi)^3} \int d^3x \, e^{i(\vec{k}' - \vec{k}) \cdot \vec{x}} V_n(|\vec{x}|) \,, \end{aligned} \tag{S79}$$

where the second line follows upon the substitution $\vec{x} - \vec{R}_n \rightarrow \vec{x}$. This depends on the position of the n-th atom through the simple phase factor $e^{i(\vec{k}' - \vec{k}) \cdot \vec{R}_n}$. The volume integral is, up to a universal constant, the scattering amplitude for scattering from $V_n(|\vec{x}|)$, in the Born approximation, at momentum transfer $\vec{k}' - \vec{k}$ (which is the momentum transferred to the scattering atom). Because of the spherical symmetry assumed, the scattering amplitude depends only on $|\vec{k}' - \vec{k}|$.

S6.1 Zeroth-order Absorption Cross Section

Next, we calculate the zeroth-order contribution to the absorption cross section in σ_{abs}, i.e.,

$$\begin{aligned}
\sigma_{\text{abs}}^{(0)} &= -\frac{8\pi\alpha}{\omega_x}\int d^3k\int d^3k'\,\text{Im}\left\{G^{(0)}(k,\mathcal{E})\delta(\vec{k}-\vec{k}')(\vec{k}\cdot\vec{\epsilon}^{\,*})\left\langle\vec{k}\middle|i\right\rangle^*(\vec{k}'\cdot\vec{\epsilon})\left\langle\vec{k}'\middle|i\right\rangle\right\} \\
&= -\frac{8\pi\alpha}{\omega_x}\text{Im}\left\{\int d^3k\,G^{(0)}(k,\mathcal{E})(\vec{k}\cdot\vec{\epsilon}^{\,*})\left\langle\vec{k}\middle|i\right\rangle^*(\vec{k}\cdot\vec{\epsilon})\left\langle\vec{k}\middle|i\right\rangle\right\} \\
&= -\frac{8\pi\alpha}{3\omega_x}\text{Im}\left\{\int d^3k\,G^{(0)}(k,\mathcal{E})k^2\left|\left\langle\vec{k}\middle|i\right\rangle\right|^2\right\}\,, \qquad \text{(S80)}
\end{aligned}$$

where we have carried out the orientational averaging, Eq. (S78), in the last line. Next, we approximate the K-shell orbital by

$$\varphi_i(\vec{x}) = \frac{1}{\sqrt{\pi/Z_{\text{eff}}^3}}e^{-Z_{\text{eff}}r}\,, \qquad \text{(S81)}$$

where $r=|\vec{x}|$ is given in atomic units. With that, we obtain

$$\left\langle\vec{k}\middle|i\right\rangle = \frac{1}{(2\pi)^{3/2}}\int d^3x\,e^{-i\vec{k}\cdot\vec{x}}\varphi_i(\vec{x}) = \frac{1}{(2\pi)^{3/2}}\frac{1}{\sqrt{\pi/Z_{\text{eff}}^3}}\frac{8\pi Z_{\text{eff}}}{(Z_{\text{eff}}^2+k^2)^2}\,. \qquad \text{(S82)}$$

We then perform the volume integral in three-dimensional $\vec{k}$ space and find

$$\sigma_{\text{abs}}^{(0)} = -\frac{8\pi\alpha}{3\omega_x}\frac{64\pi}{(2\pi)^3}Z_{\text{eff}}^5\,4\pi\,\text{Im}\left\{\int_0^\infty dk\,\frac{k^4}{(Z_{\text{eff}}^2+k^2)^4}\,G^{(0)}(k,\mathcal{E})\right\}\,. \qquad \text{(S83)}$$

In a next step, we use $\mathcal{M}(k=\sqrt{2\mathcal{E}},\mathcal{E})\equiv\mathcal{M}(\mathcal{E})$ as an approximation for the evaluation of $G^{(0)}(k,\mathcal{E})$ and that $\mathcal{M}(k,\mathcal{E})$ is an even function of k. With this, the zeroth-order Green's function becomes

$$G^{(0)}(k,\mathcal{E}) \simeq \frac{1}{\left[\mathcal{E}-\mathcal{M}(\mathcal{E})+i\frac{\Gamma_i}{2}\right]-\frac{k^2}{2}} = \frac{1}{\tilde{\mathcal{E}}(\mathcal{E})-\frac{k^2}{2}}\,, \qquad \text{(S84)}$$

where we have defined the complex-valued energy

$$\tilde{\mathcal{E}}(\mathcal{E}) = \mathcal{E} - \mathcal{M}(\mathcal{E}) + i\frac{\Gamma_i}{2}\,. \tag{S85}$$

In this approximation, $G^{(0)}(k, \mathcal{E})$ has simple poles at

$$k_{\pm} = \pm\tilde{k}(\mathcal{E}). \tag{S86}$$

Here,

$$\tilde{k}(\mathcal{E}) = \sqrt{2\tilde{\mathcal{E}}(\mathcal{E})} \tag{S87}$$

plays the role of a complex-valued momentum and is of central importance in this work.

Since, as we showed in Sec. S4.4, $\mathrm{Im}\{\mathcal{M}(\mathcal{E})\} < 0$ for the relevant energies, $\tilde{\mathcal{E}} = \mathcal{E} - \mathcal{M}(\mathcal{E}) + i\frac{\Gamma_i}{2}$ lies in the first quadrant in the complex plane, provided $\mathcal{E} - \mathrm{Re}\{\mathcal{M}(\mathcal{E})\} > 0$. As we saw in Fig. S4, $|\mathrm{Re}\{\mathcal{M}(\mathcal{E})\}| \ll \mathcal{E}$ in the entire energy range of relevance for EXAFS, i.e., the condition $\mathcal{E} - \mathrm{Re}\{\mathcal{M}(\mathcal{E})\} > 0$ is indeed satisfied. Hence, the imaginary part of the complex momentum $\tilde{k}(\mathcal{E}) = \sqrt{2\tilde{\mathcal{E}}(\mathcal{E})}$ is positive (see Fig. S6). It follows that k_+ lies above the real axis in the complex k plane, whereas k_- lies below it. Note further that within the approximation made, the integrand in Eq. (S83) is an even function of k, so that we may write

$$\int_0^{\infty} dk\, \frac{k^4}{(Z_{\mathrm{eff}}^2 + k^2)^4}\, G^{(0)}(k, \mathcal{E}) = \frac{1}{2}\int_{-\infty}^{\infty} dk\, \frac{k^4}{(Z_{\mathrm{eff}}^2 + k^2)^4}\, G^{(0)}(k, \mathcal{E})\,. \tag{S88}$$

Closing the contour in the upper half of the complex k plane, we obtain

$$\begin{aligned} & \frac{1}{2}\int_{-\infty}^{\infty} dk\, \frac{k^4}{(Z_{\mathrm{eff}}^2 + k^2)^4}\, G^{(0)}(k, \mathcal{E}) \\ = & \int_{-\infty}^{\infty} dk\, \frac{k^4}{\{(k - iZ_{\mathrm{eff}})(k + iZ_{\mathrm{eff}})\}^4}\, \frac{-1}{(k - \sqrt{2\tilde{\mathcal{E}}})(k + \sqrt{2\tilde{\mathcal{E}}})} \\ = & -\pi i \frac{\left(\sqrt{2\tilde{\mathcal{E}}}\right)^3}{\left(Z_{\mathrm{eff}}^2 + 2\tilde{\mathcal{E}}\right)^4}\,. \end{aligned} \tag{S89}$$

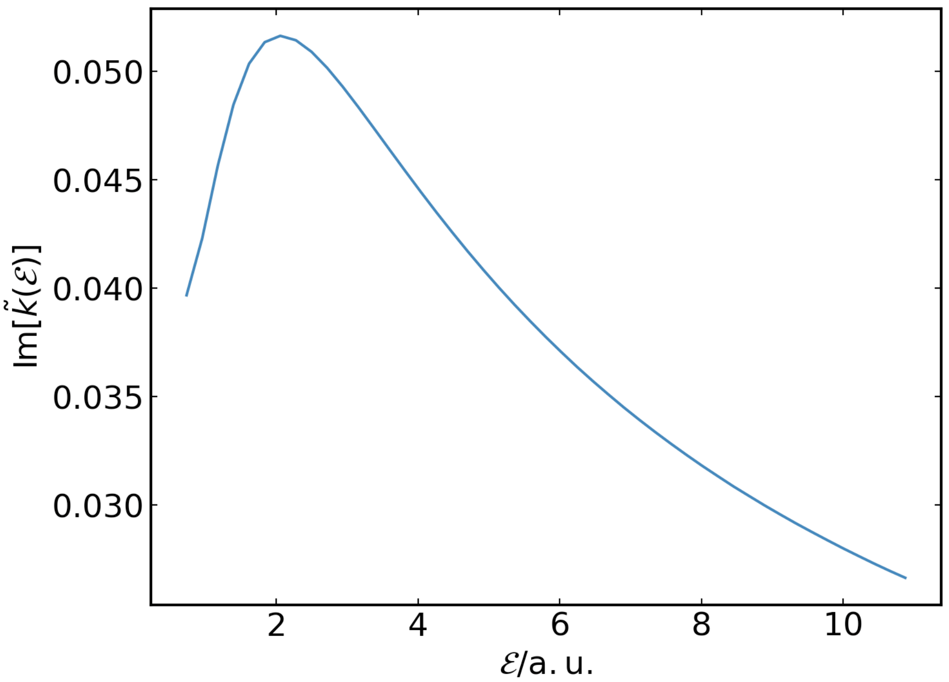

Figure S6: The imaginary part of $\tilde{k}(\mathcal{E}) = \sqrt{2\tilde{\mathcal{E}}(\mathcal{E})}$ given in atomic units, using the dissipative photoelectron self-energy of liquid water.

Note that we have omitted the contribution from the pole at $k = iZ_{\text{eff}}$: To leading nonvanishing order in $2\tilde{\mathcal{E}}/Z_{\text{eff}}^2$, the contribution from that pole to the integral in Eq. (S89) is purely real. Thus, in this approximation, which effectively neglects the imaginary part of $\tilde{\mathcal{E}}$ in comparison to the inner-shell binding energy, the contribution of the pole at iZ_{eff} to the photoabsorption cross section in Eq. (S83) vanishes. Collecting results, we find for the K-shell photoabsorption cross section to zeroth order in V_{sc}

$$\sigma_{\text{abs}}^{(0)} = \frac{256}{3}\pi\alpha\frac{Z_{\text{eff}}^5}{\omega_x}\text{Re}\left\{\frac{\left(\sqrt{2\tilde{\mathcal{E}}}\right)^3}{\left(Z_{\text{eff}}^2 + 2\tilde{\mathcal{E}}\right)^4}\right\}, \tag{S90}$$

with $\tilde{\mathcal{E}} = \mathcal{E} - \mathcal{M}(\sqrt{2\mathcal{E}}, \mathcal{E}) + i\frac{\Gamma_i}{2}$ and $\mathcal{E} = \omega_x - I_i$.

S6.2 First-order Absorption Cross Section

The contribution to the absorption cross section to first order in $V_{\rm sc}$ is [see Eq. (S38)]

$$\sigma^{(1)}_{\rm abs} = -\frac{8\pi\alpha}{\omega_x}{\rm Im}\,\{I(\mathcal{E})\}\ , \tag{S91}$$

with

$$I(\mathcal{E}) \;=\; \int d^3k\int d^3k' G^{(0)}(k,\mathcal{E})\left\langle \vec{k}\middle|V_{\rm sc}\middle|\vec{k}'\right\rangle G^{(0)}(k',\mathcal{E})(\vec{k}\cdot\vec{\epsilon}^{\,*})\left\langle\vec{k}\middle|i\right\rangle^*(\vec{k}'\cdot\vec{\epsilon})\left\langle\vec{k}'\middle|i\right\rangle . \tag{S92}$$

We use Eq. (S82) and replace the momentum vector $\vec{k}$ by the canonical momentum operator $\vec{\nabla}/i$. Moreover, with $\left\langle\vec{x}\middle|\vec{k}\right\rangle = \frac{1}{(2\pi)^{3/2}}e^{i\vec{k}\cdot\vec{x}}$, we get

$$\begin{aligned}\left\langle\vec{k}\middle|V_{\rm sc}\middle|\vec{k}'\right\rangle &= \int d^3x\int d^3x'\left\langle\vec{k}\middle|\vec{x}\right\rangle\left\langle\vec{x}\middle|V_{\rm sc}\middle|\vec{x}\,'\right\rangle\left\langle\vec{x}\,'\middle|\vec{k}\,'\right\rangle\\ &= \frac{1}{(2\pi)^3}\int d^3x\int d^3x' e^{-i\vec{k}\cdot\vec{x}}\left\langle\vec{x}\middle|V_{\rm sc}\middle|\vec{x}\,'\right\rangle e^{i\vec{k}'\cdot\vec{x}'}\\ &= \frac{1}{(2\pi)^3}\int d^3x'' V_{\rm sc}(\vec{x}'')e^{-i(\vec{k}-\vec{k}')\cdot\vec{x}''}\ .\end{aligned} \tag{S93}$$

Since $\vec{k}$ is real, we have

$$\begin{aligned}(\vec{k}\cdot\vec{\epsilon}^{\,*})\left\langle\vec{k}\middle|i\right\rangle^* &= \left((\vec{k}\cdot\vec{\epsilon})\left\langle\vec{k}\middle|i\right\rangle\right)^*\\ &= \left(\vec{\epsilon}\cdot\left\langle\vec{k}\,\middle|\vec{k}\middle|\,i\right\rangle\right)^*\\ &= \left(\vec{\epsilon}\cdot\left\langle\vec{k}\,\middle|\vec{\nabla}/i\middle|\,i\right\rangle\right)^*\\ &= \left(\left\langle\vec{k}\,\middle|\vec{\epsilon}\cdot\vec{\nabla}/i\middle|\,i\right\rangle\right)^*\ ,\end{aligned} \tag{S94}$$

so that

$$
\begin{aligned}
I(\mathcal{E}) &= \int \frac{d^3k}{(2\pi)^3} \int \frac{d^3k'}{(2\pi)^3} \int d^3x \int d^3x' \int d^3x'' G^{(0)}(k,\mathcal{E}) V_{\rm sc}(\vec{x}'') e^{-i(\vec{k}-\vec{k}')\cdot\vec{x}''} G^{(0)}(k',\mathcal{E}) \\
&\quad \times \left[\vec{\epsilon}\cdot\frac{\vec{\nabla}}{i}\varphi_i(\vec{x})\right]^* e^{i\vec{k}\cdot\vec{x}} \left[\vec{\epsilon}\cdot\frac{\vec{\nabla}'}{i}\varphi_i(\vec{x}')\right] e^{-i\vec{k}'\cdot\vec{x}'} \\
&= \int d^3x \int d^3x' \left[\vec{\epsilon}\cdot\frac{\vec{\nabla}}{i}\varphi_i(\vec{x})\right]^* \left[\vec{\epsilon}\cdot\frac{\vec{\nabla}'}{i}\varphi_i(\vec{x}')\right] \int d^3x'' V_{\rm sc}(\vec{x}'') \\
&\quad \times \int \frac{d^3k}{(2\pi)^3} \frac{1}{\tilde{\mathcal{E}} - \frac{k^2}{2}} e^{i\vec{k}\cdot(\vec{x}-\vec{x}'')} \int \frac{d^3k'}{(2\pi)^3} \frac{1}{\tilde{\mathcal{E}} - \frac{k'^2}{2}} e^{i\vec{k}'\cdot(\vec{x}''-\vec{x}')} \,. \qquad \text{(S95)}
\end{aligned}
$$

Again, $\tilde{\mathcal{E}}$ lies in the first quadrant of the complex plane and using the definition $\tilde{k} = +\sqrt{2\tilde{\mathcal{E}}}$, we see that $e^{i\tilde{k}r}$ is exponentially damped for large $r > 0$. Thus, we have for the real-space representation of the zeroth-order Green's function

$$
\begin{aligned}
\int \frac{d^3k}{(2\pi)^3} \frac{1}{\tilde{\mathcal{E}} - \frac{k^2}{2}} e^{i\vec{k}\cdot\vec{x}} &= \frac{2\pi}{(2\pi)^3} \int_0^\infty dk\, k^2 \frac{1}{\tilde{\mathcal{E}} - \frac{k^2}{2}} \int_{-1}^{+1} dy\, e^{ik|\vec{x}|y} \\
&= \frac{1}{(2\pi)^2} \int_0^\infty dk \frac{k}{\tilde{\mathcal{E}} - \frac{k^2}{2}} \frac{1}{i|\vec{x}|} e^{ik|\vec{x}|} + \frac{1}{(2\pi)^2} \int_{-\infty}^0 dk \frac{k}{\tilde{\mathcal{E}} - \frac{k^2}{2}} \frac{1}{i|\vec{x}|} e^{ik|\vec{x}|} \\
&= \frac{1}{(2\pi)^2} \int_{-\infty}^\infty dk \frac{k}{\tilde{\mathcal{E}} - \frac{k^2}{2}} \frac{e^{ik|\vec{x}|}}{i|\vec{x}|} \\
&= -\frac{1}{2\pi} \frac{e^{i\tilde{k}|\vec{x}|}}{|\vec{x}|} \,. \qquad \text{(S96)}
\end{aligned}
$$

We have closed the contour in the upper half of the complex k plane.

It follows that

$$
\begin{aligned}
I(\mathcal{E}) &= \frac{1}{(2\pi)^2} \int d^3x \int d^3x' \left[\vec{\epsilon}\cdot\frac{\vec{\nabla}}{i}\varphi_i(\vec{x})\right]^* \left[\vec{\epsilon}\cdot\frac{\vec{\nabla}'}{i}\varphi_i(\vec{x}')\right] \\
&\quad \times \int d^3x'' V_{\rm sc}(\vec{x}'') \frac{e^{i\tilde{k}|\vec{x}''-\vec{x}|}}{|\vec{x}''-\vec{x}|} \frac{e^{i\tilde{k}|\vec{x}''-\vec{x}'|}}{|\vec{x}''-\vec{x}'|} \,. \qquad \text{(S97)}
\end{aligned}
$$

No particular simplification seems possible for the term with $n = 0$ in $V_{\rm sc}$. However, for a term with $n \neq 0$, we can use that $|\vec{x}|$ and $|\vec{x}'|$ are constrained by the spatial extension of the

inner-shell orbital to a region near the origin with a length scale of order $1/Z_{\text{eff}}$ in atomic units. In contrast, the vector $|\vec{x}''|$ is constrained by $V_n(|\vec{x}'' - \vec{R}_n|)$ to lie in the vicinity of $\vec{R}_n$. Hence,

$$\begin{aligned} |\vec{x}'' - \vec{x}| &= |\vec{x}'' - \vec{R}_n - \vec{x} + \vec{R}_n| \\ &= \sqrt{\left(\vec{x}'' - \vec{R}_n - \vec{x}\right)^2 + 2\left(\vec{x}'' - \vec{R}_n - \vec{x}\right)\cdot\vec{R}_n + R_n^2} \\ &\simeq R_n\left\{1 + \frac{1}{R_n^2}\left(\vec{x}'' - \vec{R}_n - \vec{x}\right)\cdot\vec{R}_n\right\} . \end{aligned} \tag{S98}$$

Therefore, for $n \neq 0$, we have

$$\begin{aligned} I_n(\mathcal{E}) &\simeq \frac{1}{(2\pi)^2}\int d^3x \int d^3x' \left[\vec{\epsilon}\cdot\frac{\vec{\nabla}}{i}\varphi_i(\vec{x})\right]^* \left[\vec{\epsilon}\cdot\frac{\vec{\nabla}'}{i}\varphi_i(\vec{x}')\right] \\ &\quad \times \int d^3x'' V_n(|\vec{x}'' - \vec{R}_n|)\frac{e^{i\tilde{k}|\vec{x}''-\vec{x}|}}{|\vec{x}''-\vec{x}|}\frac{e^{i\tilde{k}|\vec{x}''-\vec{x}'|}}{|\vec{x}''-\vec{x}'|} \\ &\simeq \frac{1}{(2\pi)^2}\frac{1}{R_n^2}e^{2i\tilde{k}R_n}\int d^3x \int d^3x' \left[\vec{\epsilon}\cdot\frac{\vec{\nabla}}{i}\varphi_i(\vec{x})\right]^* \left[\vec{\epsilon}\cdot\frac{\vec{\nabla}'}{i}\varphi_i(\vec{x}')\right] \\ &\quad \times \int d^3x'' V_n(|\vec{x}'' - \vec{R}_n|) e^{i\tilde{k}\frac{\vec{R}_n}{R_n}\cdot\left(\vec{x}''-\vec{R}_n-\vec{x}\right)} e^{i\tilde{k}\frac{\vec{R}_n}{R_n}\cdot\left(\vec{x}''-\vec{R}_n-\vec{x}'\right)} . \end{aligned} \tag{S99}$$

We recognize that in this approximation, $I_n(\mathcal{E})$ depends on the first-order Born amplitude for electron backward-scattering from V_n:

$$f_n^{(1)}\left(-\tilde{k}\frac{\vec{R}_n}{R_n}, \tilde{k}\frac{\vec{R}_n}{R_n}\right) = -\frac{1}{2\pi}\int d^3x'' e^{i\tilde{k}\frac{\vec{R}_n}{R_n}\cdot\vec{x}''} V_n(|\vec{x}''|) e^{i\tilde{k}\frac{\vec{R}_n}{R_n}\cdot\vec{x}''} . \tag{S100}$$

Note, however, that we must evaluate the scattering amplitude at complex momenta ($\tilde{k} \in \mathbb{C}$). Similarly, $I_n(\mathcal{E})$ depends on transition dipole matrix elements between the inner-shell orbital

and plane-wave states involving $\tilde{k} \in \mathbb{C}$. Hence,

$$
\begin{aligned}
I_n(\mathcal{E}) \;\simeq\; & \frac{1}{(2\pi)^2}\frac{1}{R_n^2}e^{2i\tilde{k}R_n}(-2\pi)f_n^{(1)}\left(-\tilde{k}\frac{\vec{R}_n}{R_n},\tilde{k}\frac{\vec{R}_n}{R_n}\right) \\
& \times(2\pi)^3\int d^3x\left[\frac{1}{(2\pi)^{3/2}}e^{i\tilde{k}^*\frac{\vec{R}_n}{R_n}\cdot\vec{x}}\vec{\epsilon}\cdot\frac{\vec{\nabla}}{i}\varphi_i(\vec{x})\right]^*\int d^3x'\frac{1}{(2\pi)^{3/2}}e^{-i\tilde{k}\frac{\vec{R}_n}{R_n}\cdot\vec{x}'}\vec{\epsilon}\cdot\frac{\vec{\nabla}'}{i}\varphi_i(\vec{x}')\,.
\end{aligned}
\tag{S101}
$$

Everywhere $\tilde{k} = \sqrt{2\tilde{\mathcal{E}}}$ appears, the inclusion of the electron self-energy makes a difference. The product of the two transition dipoles in the second line of Eq. (S101) is related to $\sigma_{\text{abs}}^{(0)}$.

S7 Determination of the Relative EXAFS Signal

The first-order contribution to the photoabsorption cross section is a sum over atomic contributions:

$$
\sigma_{\text{abs}}^{(1)} = \sum_n \sigma_n^{(1)}\,. \tag{S102}
$$

The term $\sigma_0^{(1)}$ is a correction to the cross section from the atomic potential of the absorbing atom. Thus, in the current approximation, $\sigma_{\text{abs}}^{(0)} + \sigma_0^{(1)}$ is the atomic photoabsorption cross section in the absence of photoelectron scattering from other atoms. Hence, what is usually called χ in the EXAFS literature is given, to leading order, by

$$
\begin{aligned}
\chi &= \frac{\sigma_{\text{abs}} - [\sigma_{\text{abs}}^{(0)} + \sigma_0^{(1)}]}{\sigma_{\text{abs}}^{(0)} + \sigma_0^{(1)}} \\
&= \frac{\sum_{n\neq 0}\sigma_n^{(1)}}{\sigma_{\text{abs}}^{(0)} + \sigma_0^{(1)}} \\
&\approx \frac{1}{\sigma_{\text{abs}}^{(0)}}\sum_{n\neq 0}\sigma_n^{(1)}\,.
\end{aligned}
\tag{S103}
$$

Note that keeping $\sigma_0^{(1)}$ would give a second-order contribution. For $n \neq 0$, $\sigma_n^{(1)}$ follows from Eqs. (S91) and (S101) as

$$
\sigma_n^{(1)} = -\frac{8\pi\alpha}{\omega_x}\left(-\frac{1}{2\pi R_n^2}\right)\text{Im}\left\{e^{2i\tilde{k}R_n}f_n^{(1)}D_1D_2^*\right\}\,, \tag{S104}
$$

where

$$D_1 = \int d^3x\, e^{-i\tilde{k}\frac{\vec{R}_n}{R_n}\cdot\vec{x}}\vec{\epsilon}\cdot\frac{\vec{\nabla}}{i}\varphi_i(\vec{x})\,, \tag{S105}$$

and

$$D_2 = \int d^3x\, e^{i\tilde{k}^*\frac{\vec{R}_n}{R_n}\cdot\vec{x}}\vec{\epsilon}\cdot\frac{\vec{\nabla}}{i}\varphi_i(\vec{x})\,. \tag{S106}$$

Focusing on D_1, and exploiting that $\varphi_i(\vec{x})$ decays faster as a function of $|\vec{x}|$ than $e^{-i\tilde{k}\frac{\vec{R}_n}{R_n}\cdot\vec{x}}$ may be expected to diverge, we obtain after an integration by parts

$$\begin{aligned}
D_1 &= -\frac{1}{i}\int d^3x\,\varphi_i(\vec{x})\vec{\epsilon}\cdot\vec{\nabla}e^{-i\tilde{k}\frac{\vec{R}_n}{R_n}\cdot\vec{x}} \\
&= \tilde{k}\vec{\epsilon}\cdot\frac{\vec{R}_n}{R_n}\int d^3x\,\varphi_i(\vec{x})e^{-i\tilde{k}\frac{\vec{R}_n}{R_n}\cdot\vec{x}} \\
&= \sqrt{\frac{Z_{\text{eff}}^3}{\pi}}\tilde{k}\vec{\epsilon}\cdot\frac{\vec{R}_n}{R_n}\int d^3x\,e^{-Z_{\text{eff}}r}e^{-i\tilde{k}\frac{\vec{R}_n}{R_n}\cdot\vec{x}} \\
&= \sqrt{\frac{Z_{\text{eff}}^3}{\pi}}\tilde{k}\vec{\epsilon}\cdot\frac{\vec{R}_n}{R_n}2\pi\int_0^\infty dr\,r^2e^{-Z_{\text{eff}}r}\frac{1}{-i\tilde{k}r}\left[e^{-i\tilde{k}r}-e^{i\tilde{k}r}\right] \\
&= 8\sqrt{\pi Z_{\text{eff}}^5}\vec{\epsilon}\cdot\frac{\vec{R}_n}{R_n}\frac{\tilde{k}}{\left(Z_{\text{eff}}^2+\tilde{k}^2\right)^2}.
\end{aligned} \tag{S107}$$

D_2 follows immediately by replacing $\tilde{k}$ in D_1 with $-\tilde{k}^*$, i.e.,

$$D_2 = 8\sqrt{\pi Z_{\text{eff}}^5}\vec{\epsilon}\cdot\frac{\vec{R}_n}{R_n}\frac{-\tilde{k}^*}{\left(Z_{\text{eff}}^2+\tilde{k}^{*2}\right)^2}. \tag{S108}$$

Hence,

$$\begin{aligned}
D_1D_2^* &= -64\pi Z_{\text{eff}}^5\left(\vec{\epsilon}\cdot\frac{\vec{R}_n}{R_n}\right)^2\frac{\tilde{k}^2}{\left(Z_{\text{eff}}^2+\tilde{k}^2\right)^4} \\
&= -\frac{64}{3}\pi Z_{\text{eff}}^5\frac{\tilde{k}^2}{\left(Z_{\text{eff}}^2+\tilde{k}^2\right)^4}\,.
\end{aligned} \tag{S109}$$

In the last line, we have carried out orientational averaging using

$$\frac{1}{4\pi}\int d\Omega\left(\vec{\epsilon}\cdot\frac{\vec{R}_n}{R_n}\right)^2=\frac{1}{3}\,. \tag{S110}$$

Finally, we obtain

$$\sigma_n^{(1)}=-\frac{256}{3}\frac{\pi\alpha}{\omega_x R_n^2}Z_{\text{eff}}^5\,\text{Im}\left\{e^{2i\tilde{k}R_n}f_n^{(1)}\frac{\tilde{k}^2}{\left(Z_{\text{eff}}^2+\tilde{k}^2\right)^4}\right\}, \tag{S111}$$

and, thus, using Eqs. (S90) and (S103)

$$\begin{aligned}\chi &= -\sum_{n\neq 0}\frac{1}{R_n^2}\frac{\text{Im}\left\{e^{2i\tilde{k}R_n}f_n^{(1)}\frac{\tilde{k}^2}{\left(Z_{\text{eff}}^2+\tilde{k}^2\right)^4}\right\}}{\text{Re}\left\{\frac{\tilde{k}^3}{\left(Z_{\text{eff}}^2+\tilde{k}^2\right)^4}\right\}}\\ &= \sum_{n\neq 0}\frac{1}{R_n^2}\frac{\text{Im}\left\{e^{i(2\tilde{k}R_n+\pi)}f_n^{(1)}\frac{\tilde{k}^2}{\left(Z_{\text{eff}}^2+\tilde{k}^2\right)^4}\right\}}{\text{Re}\left\{\frac{\tilde{k}^3}{\left(Z_{\text{eff}}^2+\tilde{k}^2\right)^4}\right\}}\,.\end{aligned} \tag{S112}$$

In the last line, we have incorporated the minus sign in the exponent, reflecting an overall phase shift of π of the reflected photoelectron wave function relative to the initial one.

We now see that in our framework, in which $\tilde{k}\in\mathbb{C}$, the zeroth-order contribution $\sigma_{\text{abs}}^{(0)}$ cannot be fully eliminated from χ. This reflects the impact of the dielectric medium, through a phase shift and damping, on the wave function of the photoelectron before it interacts with the scattering atom (within the mean-field picture used). Furthermore, through

$$\tilde{k}(\mathcal{E})=\sqrt{2\tilde{\mathcal{E}}(\mathcal{E})}=\sqrt{2\left(\mathcal{E}-\mathcal{M}(\mathcal{E})+i\frac{\Gamma_i}{2}\right)} \tag{S113}$$

in $e^{2i\tilde{k}R_n}$ in Eq. (S112), both the real part and the imaginary part of the photoelectron self-energy contribute to the oscillatory structure of the EXAFS signal, and its decay, as a

function of

$$k = \sqrt{2\mathcal{E}} = \sqrt{2(\omega_x - I_i)}\,. \tag{S114}$$

A related consideration is the following: In the absence of a dielectric solvent and neglecting core-hole decay, $\tilde{k}$ is real. Owing to the spherical symmetry assumed for the atomic scattering potential V_n, the photoelectron scattering amplitude $f_n^{(1)}$ [Eq. (S100)] is then also real. Hence, in this special case, Eq. (S112) may be simplified as follows:

$$\begin{aligned}
\chi &= \sum_{n\neq 0} \frac{1}{R_n^2} \frac{\mathrm{Im}\left\{ e^{i(2\tilde{k}R_n+\pi)} f_n^{(1)} \frac{\tilde{k}^2}{\left(Z_{\mathrm{eff}}^2+\tilde{k}^2\right)^4} \right\}}{\mathrm{Re}\left\{ \frac{\tilde{k}^3}{\left(Z_{\mathrm{eff}}^2+\tilde{k}^2\right)^4} \right\}} \\
&= \sum_{n\neq 0} \frac{1}{R_n^2} f_n^{(1)} \frac{\frac{\tilde{k}^2}{\left(Z_{\mathrm{eff}}^2+\tilde{k}^2\right)^4}}{\frac{\tilde{k}^3}{\left(Z_{\mathrm{eff}}^2+\tilde{k}^2\right)^4}} \mathrm{Im}\left\{ e^{i(2\tilde{k}R_n+\pi)} \right\} \\
&= \sum_{n\neq 0} \frac{1}{\tilde{k}R_n^2} f_n^{(1)} \sin\!\left(2\tilde{k}R_n + \pi\right).
\end{aligned} \tag{S115}$$

This is a simplified version of the familiar EXAFS equation for a p-wave photoelectron. In this case, we have a k-independent phase shift. Once the solvent and the decay of the core hole are included, $\tilde{k}$ becomes complex, which generates an imaginary part in $f_n^{(1)}$. An additional k-dependent phase shift then appears in the sine function. This also shows from another perspective the influence of a dielectric bath on the oscillation in EXAFS.

S7.1 Numerical Evaluation of the EXAFS Scattering Amplitude

In order to evaluate the photoelectron scattering amplitude $f_n^{(1)}$ [Eq. (S100)] for each scattering atomic species, we utilize XATOM[S15–S17] to compute the corresponding atomic potentials V_n in Hartree-Fock-Slater approximation. (Since the photoelectron may scatter from any electron in a scattering atom, the Latter correction[S18] available in XATOM must not be used.) Exploiting the spherical symmetry of V_n, the angular integral in Eq. (S100) may be carried out analytically; the remaining radial integral we perform numerically, for each

$\tilde{k} \in \mathbb{C}$ considered.

Figures S7 and S8 show the resulting real and imaginary parts of the scattering amplitude of oxygen (O) and hydrogen (H), respectively, as a function of the complex momentum $\tilde{k}$. In both figures, the real part of $\tilde{k}$ lies between 2 a.u. and 7 a.u., corresponding to photoelectron kinetic energies approximately between 50 and 650 eV.

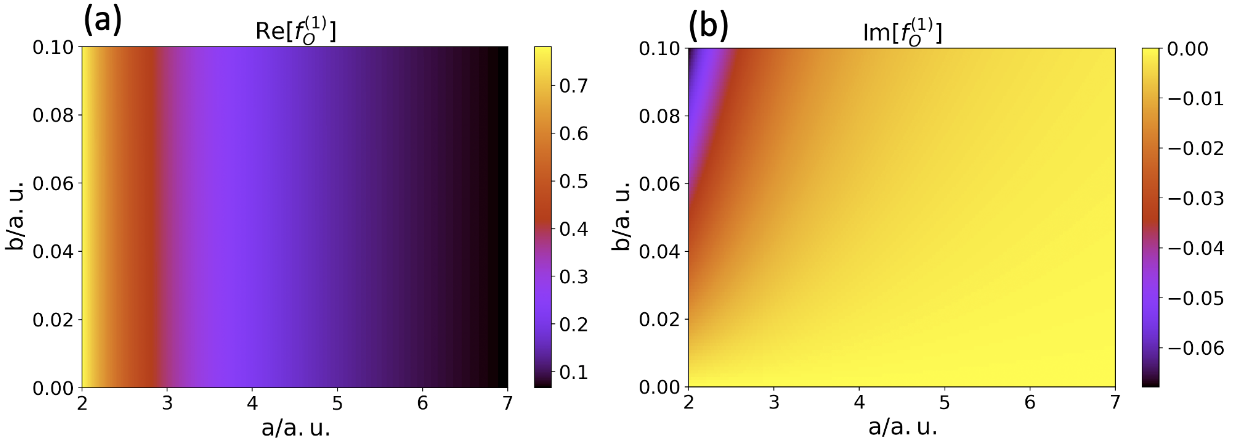

Figure S7: Real and imaginary parts of the scattering amplitude $f^{(1)}$ for the oxygen atom in atomic units, with $a = \text{Re}\ \tilde{k}$ and $b = \text{Im}\ \tilde{k}$.

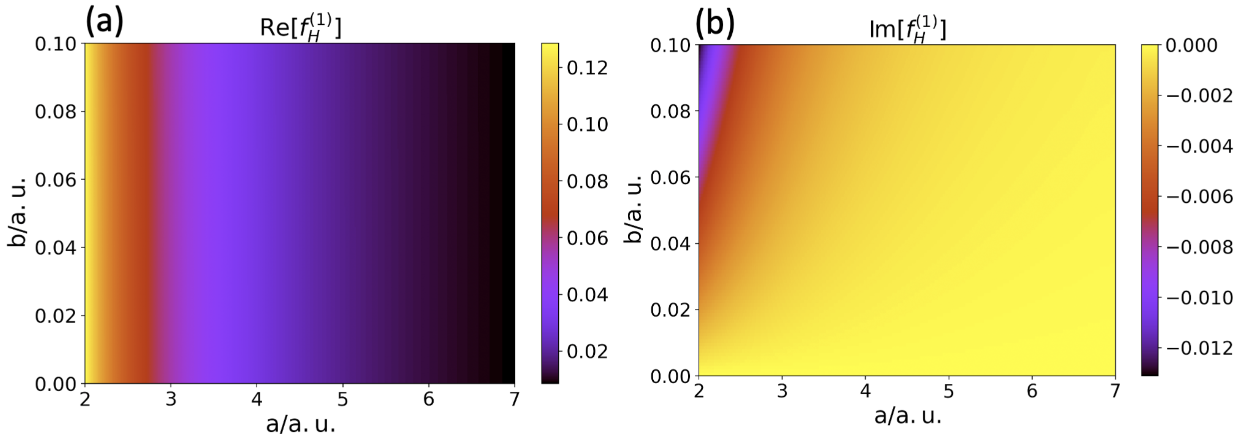

Figure S8: Real and imaginary parts of the scattering amplitude $f^{(1)}$ for the hydrogen atom in atomic units, with $a = \text{Re}\ \tilde{k}$ and $b = \text{Im}\ \tilde{k}$.

If the dielectric is not considered and we neglect core-hole decay, $\tilde{k}$ is a real number. Then $f_n^{(1)}$ is also real. In Fig. S9, we show the scattering amplitude of O for this situation.

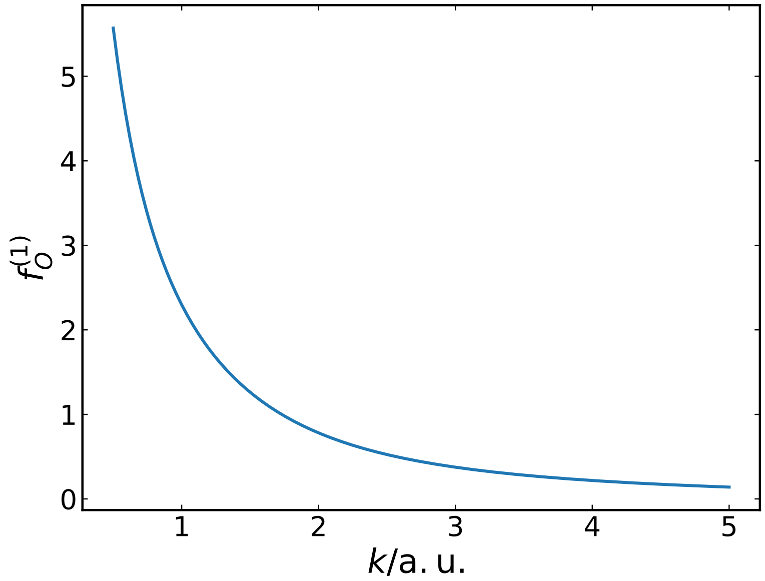

Figure S9: Scattering amplitude $f^{(1)}$ for the oxygen atom in the absence of a dielectric solvent and neglecting core-hole decay, as a function of the real k in atomic units.

S7.2 Additional Phase Shifts

Equation (S97) describes the ejection of a photoelectron from the atom absorbing the x-ray photon and the subsequent scattering of the photoelectron from the individual atoms in the environment of the absorbing atom. Up to a constant, each of the two functions of the form $e^{i\tilde{k}|\vec{x}''-\vec{x}|}/|\vec{x}''-\vec{x}|$ in Eq. (S97) is a real-space representation of the Green's function of an electron in a homogeneous dielectric medium [see Eq. (S96)]. Such a function does not take into consideration the modification of the photoelectron wave in the presence of the various atomic scattering potentials. Within the absorbing atom and within the scattering atoms, the atomic potentials are so strong that neglecting the nonperturbative modification of the photoelectron wave function within those volumes is too inaccurate.

Hence, we introduce phase shifts ϕ_{abs} and ϕ_{back} into Eq. (S112). ϕ_{abs} is intended to capture the impact of the atomic potential of the absorbing atom on the photoelectron. Not only does a p-wave photoelectron wave function experience a universal phase shift of $\pi/2$ (or, more precisely, $-\pi/2$), but as a consequence of the atomic potential V_0 of the absorbing atom,

it also experiences an additional p-wave phase shift of δ_1. Since XATOM provides access to atomic continuum wave functions, determining δ_1 is straightforward using XATOM. In the interference term between the direct and indirect pathways taken by the photoelectron, the atomic phase shift δ_1 gives rise to an overall phase shift of $\phi_{\text{abs}} = 2\delta_1$. [The overall universal p-wave phase shift of $2 \times \pi/2 = \pi$ is already included in the exponential function in the second line of Eq. (S112).] The phase shift ϕ_{back} is the phase argument of the numerically exact amplitude for photoelectron backward scattering from the n-th scattering atom, i.e.,

$$\phi_{\text{back}} = \arg\left[\sum_l (-1)^l (2l+1) \frac{e^{i\delta_l}}{k} \sin(\delta_l)\right]. \tag{S116}$$

The required partial-wave phase shifts δ_l for a given atomic potential V_n ($n \neq 0$) we also compute using XATOM continuum wave functions. The results are shown in Fig. S10.

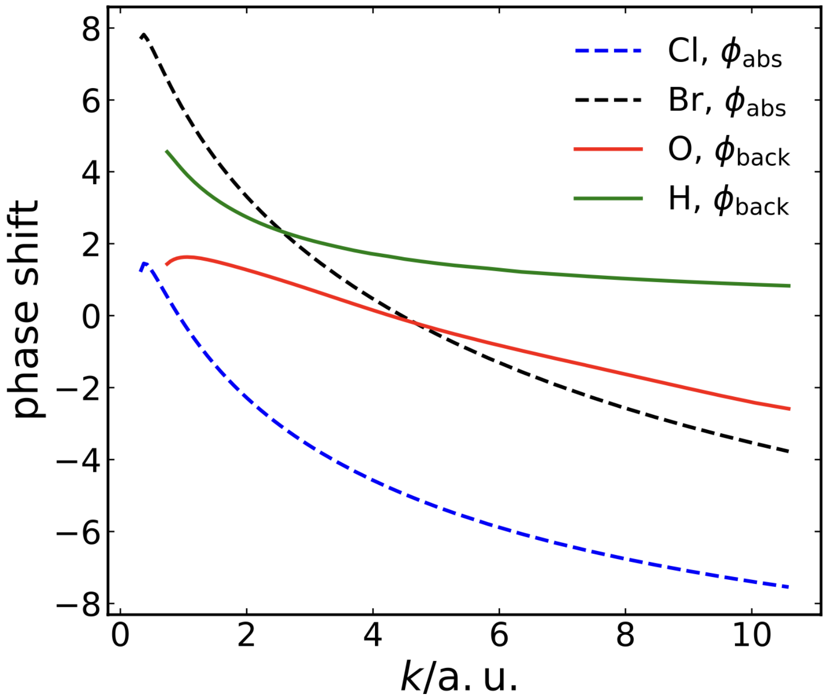

Figure S10: Phase shifts ϕ_{abs} (dashed lines) for atomic absorbers Cl (blue) and Br (black) and ϕ_{back} (solid lines) for atomic scatterers O (red) and H (green), calculated using XATOM.

In summary, after integrating the atomic phase shifts ϕ_{abs} and ϕ_{back} in Eq. (S112), our

working equation reads

$$\chi = \sum_{n\neq 0} \frac{1}{R_n^2} \frac{\mathrm{Im}\left\{ e^{i(2\tilde{k}R_n+\pi)} f_n^{(1)} e^{i(\phi_{\mathrm{abs}}+\phi_{\mathrm{back}})} \frac{\tilde{k}^2}{\left(Z_{\mathrm{eff}}^2+\tilde{k}^2\right)^4} \right\}}{\mathrm{Re}\left\{ \frac{\tilde{k}^3}{\left(Z_{\mathrm{eff}}^2+\tilde{k}^2\right)^4} \right\}} \,. \tag{S117}$$

This is Eq. (13) in the main text.

References

[S1] Santra, R. Concepts in x-ray physics, *J. Phys. B: At. Mol. Opt. Phys.* **2009**, 42, 023001.

[S2] Buth C.; Santra, R. Theory of x-ray absorption by laser-dressed atoms, *Phys. Rev. A* **2007**, 75, 033412.

[S3] Fetter, A. L.; Walecka, J. D. *Quantum theory of many-particle systems*; McGraw-Hill: New York, 1971.

[S4] Hedin, L. New method for calculating the one-particle Green's function with application to the electron-gas problem, *Phys. Rev.* **1965**, 139, A796 - A823.

[S5] Hedin L.; Lundqvist, S. Effects of electron-electron and electron-phonon interactions on the one-electron states of solids, *Solid State Physics* **1969**, 23, 1 - 181.

[S6] Emfietzoglou, D.; Cucinotta, F. A.; Nikjoo, H. A complete dielectric response model for liquid water: A solution of the Bethe ridge problem, *Radiat. Res.* **2005**, 164, 202 - 211.

[S7] Emfietzoglou, D.; Nikjoo, H. The effect of model approximations on single-collision distributions of low-energy electrons in liquid water, *Radiat. Res.* **2005**, 163, 98 - 111.

[S8] Emfietzoglou, D.; Papamichael, G.; Nikjoo, H. Monte Carlo electron track structure calculations in liquid water using a new model dielectric response function, *Radiat. Res.* **2017**, 188, 355 - 368.

[S9] Dingfelder, M.; Hantke, D.; Inokuti, M.; Paretzke, H.G. Electron inelastic-scattering cross sections in liquid water, *Radiat. Phys. Chem.* **1998**, 53, 1 - 18.

[S10] Hayashi H.; Hiraoka, N. Accurate measurements of dielectric and optical functions of liquid water and liquid benzene in the VUV region (1-100 eV) using small-angle inelastic x-ray scattering, *J. Phys. Chem. B* **2015**, 119, 5609 - 5623.

[S11] Crowley, B.J.B. Generalized plasmon-pole approximation for the dielectric function of a multicomponent plasma, *arXiv:1508.05606,* **2015**; https://doi.org/10.48550/arXiv.1508.05606

[S12] Winter, B.; Weber, R.; Widdra, W.; Dittmar, M.; Faubel, M.; Hertel, I.V. Full valence band photoemission from liquid water using EUV synchrotron radiation, *J. Phys. Chem. A* **2004**, 108, 2625 - 2632 (2004).

[S13] Kas, J.J.; Sorini, A.P.; Prange, M.P.; Cambell, L.W.; Soininen, J.A.; Rehr, J.J. Many-pole model of inelastic losses in x-ray absorption spectra, *Phys. Rev. B* **2007**, 76, 195116.

[S14] Nguyen-Truong, H. T. Low-energy electron inelastic mean free paths for liquid water, *J. Phys. Condens. Matter* **2018**, 30, 155101.

[S15] Son, S.-K. ; Young, L.; Santra, R. Impact of hollow-atom formation on coherent x-ray scattering at high intensity, *Phys. Rev. A* **2011**, 83, 033402.

[S16] Jurek, Z.; Son, S.-K.; Ziaja, B.; Santra, R. XMDYN and XATOM: versatile simulation tools for quantitative modeling of x-ray free-electron laser induced dynamics of matter, *J. Appl. Cryst.* **2016**, 49, 1048 - 1056.

[S17] Inhester, L.; Jurek, Z.; Son, S.-K.; Abdullah, M. M.; Santra, R. XRAYPAC—a software package for modeling x-ray-induced dynamics of matter (2025), Version 2.1.0, GitLab `https://gitlab.desy.de/CDT/xraypac`.

[S18] Band I. M.; Trzhaskovskaya, M. B. On Latter's correction in the Hartree-Fock-Slater method, *Phys. Lett. A* **1972**, 38, 51 - 52 (1972).
